# Exact Expressions for Kullback–Leibler Divergence for Multivariate and Matrix-Variate Distributions

**DOI:** 10.3390/e26080663

**Published:** 2024-08-04

**Authors:** Victor Nawa, Saralees Nadarajah

**Affiliations:** 1 Department of Mathematics and Statistics, University of Zambia, Lusaka 10101, Zambia; vnawa@yahoo.com; 2 Department of Mathematics, University of Manchester, Manchester M13 9PL, UK

**Keywords:** beta function, gamma function, matrix-variate beta function, matrix-variate confluent hypergeometric function, matrix-variate gamma function, matrix-variate Gauss hypergeometric function, matrix-variate type I Dirichlet density, type I Dirichlet density

## Abstract

The Kullback–Leibler divergence is a measure of the divergence between two probability distributions, often used in statistics and information theory. However, exact expressions for it are not known for multivariate or matrix-variate distributions apart from a few cases. In this paper, exact expressions for the Kullback–Leibler divergence are derived for over twenty multivariate and matrix-variate distributions. The expressions involve various special functions.

## 1. Introduction

The Kullback–Leibler divergence (KLD) due to [1] is a fundamental concept in information theory and statistics used to measure the divergence between two probability distributions. It quantifies how one probability distribution diverges from a second, reference probability distribution. Specifically, it calculates the expected extra amount of information required to represent data sampled from one distribution using a code optimized for another distribution. The KLD is asymmetric and not a true metric as it does not satisfy the triangle inequality. It is widely employed in various fields including machine learning, where it serves as a key component in tasks such as model comparison, optimization, and generative modeling, providing a measure of dissimilarity or discrepancy between probability distributions [2].

Suppose X is a continuous vector-variate random variable or a continuous matrix-variate random variable having one of two probability density functions $f_{i}\left(\cdot ;\theta_{i}\right)$, i=1,2 parameterized by $\theta_{i}$, i=1,2. The KLD between $f_{1}\left(\cdot ;\theta_{1}\right)$ and $f_{2}\left(\cdot ;\theta_{2}\right)$ is defined by

(1) $$\begin{array}{c} KLD=E\left[\log\frac{f_{1}\left(X;\theta_{1}\right)}{f_{2}\left(X;\theta_{2}\right)}\right], \end{array}$$

where the expectation is with respect to $f_{1}\left(\cdot ;\theta_{1}\right)$.

Because of the increasing applications of the KLD, it is useful to have exact expressions for (1). Apart from the multivariate normal distribution, not many expressions have been derived for (1) for multivariate or matrix-variate distributions. The KLD for the multivariate generalized Gaussian distribution was derived only in 2019, see [3]. The KLD for the multivariate Cauchy distribution was derived only in 2022, see [4]. The KLD for the multivariate *t* distribution was derived only in 2023, see [5].

The aim of this paper is to derive exact expressions for (1) for over twenty multivariate and matrix-variate distributions. The exact expressions for multivariate distributions are presented in Section 2. The exact expressions for matrix-variate distributions are presented in Section 3. The derivations of all of the expressions including a technical lemma needed for the derivations are presented in Section 4. The distributions considered in this paper are continuous. We shall not be considering discrete distributions including mixtures.

The functions and parameters used in this paper are all real-valued. The calculations involve several real-valued special functions listed in the Appendix A.

## 2. Exact Expressions for Multivariate Distributions

In this section, we state the exact expressions for (1) for Dirichlet, multivariate generalized Gaussian, inverted Dirichlet, multivariate Gauss hypergeometric, multivariate Kotz type, ref. [6]’s multivariate logistic, ref. [7]’s multivariate logistic, ref. [8]’s multivariate normal, multivariate Pearson type II, multivariate Selberg beta, multivariate weighted exponential and von Mises distributions.

A closed form for (1) for the multivariate generalized Gaussian distribution was derived by [3]. But it involved a special function defined as a (p−1) folded infinite sum. The expression we give in Section 2.2 is much simpler in that it involves a single infinite sum. A closed form for (1) for the Dirichlet distribution is available in [9] and https://statproofbook.github.io/P/dir-kl.html (accessed on 1 July 2024).

### 2.1. Dirichlet Distribution

Consider the joint probability density functions

$$\begin{array}{c} f_{1}\left(x\right)=\frac{\prod_{i=1}^{K}x_{i}^{a_{i}-1}}{B\left(a_{1},\ldots ,a_{K-1};a_{K}\right)} \end{array}$$

and

$$\begin{array}{c} f_{2}\left(x\right)=\frac{\prod_{i=1}^{K}x_{i}^{b_{i}-1}}{B\left(b_{1},\ldots ,b_{K-1};b_{K}\right)} \end{array}$$

for K≥2, $a_{1}>0,\ldots ,a_{K}>0$, $b_{1}>0,\ldots ,b_{K}>0$, $0\leq x_{1}\leq 1,\ldots ,0\leq x_{K}\leq 1$ and $x_{1}+\cdots +x_{K}=1$. The corresponding KLD is

$$\begin{array}{c} KLD=\log\left[\frac{B\left(b_{1},\ldots ,b_{K-1};b_{K}\right)}{B\left(a_{1},\ldots ,a_{K-1};a_{K}\right)}\right]+\sum_{i=1}^{K}\left(a_{i}-b_{i}\right)\psi \left(a_{i}\right)-\psi \left(a_{1}+\cdots +a_{K}\right)\sum_{i=1}^{K}\left(a_{i}-b_{i}\right). \end{array}$$

### 2.2. Multivariate Generalized Gaussian Distribution ([10], p. 215)

Consider the joint probability density functions

$$\begin{array}{c} f_{1}\left(x_{1},\ldots ,x_{p}\right)=\frac{\alpha \Gamma \left(\frac{p}{2}\right)}{2\pi^{\frac{p}{2}}\Gamma \left(\frac{p}{\alpha }\right)}{\left|V_{1}\right|}^{-\frac{1}{2}}\exp\left\{-{\left(x^{T}V_{1}^{-1}x\right)}^{\frac{\alpha }{2}}\right\} \end{array}$$

and

$$\begin{array}{c} f_{2}\left(x_{1},\ldots ,x_{p}\right)=\frac{\beta \Gamma \left(\frac{p}{2}\right)}{2\pi^{\frac{p}{2}}\Gamma \left(\frac{p}{\beta }\right)}{\left|V_{2}\right|}^{-\frac{1}{2}}\exp\left\{-{\left(x^{T}V_{2}^{-1}x\right)}^{\frac{\beta }{2}}\right\} \end{array}$$

for $-\infty <x_{1}<\infty ,\ldots ,-\infty <x_{p}<\infty$, α>0, β>0 and $V_{1}$, $V_{2}$ positive definite symmetric matrices. The corresponding KLD is

KLD=logαΓpββΓpα+12logV2V1−pα+Γp2Γp+β2πp2Γpα∑r1=0∞∑r2=0r1⋯∑rp−1=0rp−2β2r1×r1r2⋯rp−2rp−1λ1β2−r1∏j=1p−1λj+1−λjrj−rj+1Brj+p−j2,12

provided that the infinite sum converges.

### 2.3. Inverted Dirichlet Distribution

Consider the joint probability density functions

$$\begin{array}{c} f_{1}\left(x\right)=\frac{\Gamma \left(a_{1}+\cdots +a_{K+1}\right)}{\Gamma \left(a_{1}\right)\cdots \Gamma \left(a_{K+1}\right)}{\left(1+\sum_{i=1}^{K}x_{i}\right)}^{-a_{1}-\cdots -a_{K+1}}\prod_{i=1}^{K}x_{i}^{a_{i}-1} \end{array}$$

and

$$\begin{array}{c} f_{2}\left(x\right)=\frac{\Gamma \left(b_{1}+\cdots +b_{K+1}\right)}{\Gamma \left(b_{1}\right)\cdots \Gamma \left(b_{K+1}\right)}{\left(1+\sum_{i=1}^{K}x_{i}\right)}^{-b_{1}-\cdots -b_{K+1}}\prod_{i=1}^{K}x_{i}^{b_{i}-1} \end{array}$$

for K≥2, $a_{1}>0,\ldots ,a_{K+1}>0$, $b_{1}>0,\ldots ,b_{K+1}>0$ and $x_{1}>0,\ldots ,x_{K}>0$. The corresponding KLD is

$$\begin{array}{cc} \; & KLD=\log\left[\frac{\Gamma \left(a_{1}+\cdots +a_{K+1}\right)\Gamma \left(b_{1}\right)\cdots \Gamma \left(b_{K+1}\right)}{\Gamma \left(b_{1}+\cdots +b_{K+1}\right)\Gamma \left(a_{1}\right)\cdots \Gamma \left(a_{K+1}\right)}\right]+\sum_{i=1}^{K}\left(a_{i}-b_{i}\right)\psi \left(a_{i}\right)\\ \; & \;\;\;-\psi \left(a_{K+1}\right)\sum_{i=1}^{K}\left(a_{i}-b_{i}\right)\\ \; & \;\;\;+\left(b_{1}+\cdots +b_{K+1}-a_{1}-\cdots -a_{K+1}\right)\left[\psi \left(a_{1}+\cdots +a_{K+1}\right)-\psi \left(a_{K+1}\right)\right]. \end{array}$$

### 2.4. Multivariate Gauss Hypergeometric Distribution [11]

Consider the joint probability density functions

$$\begin{array}{c} f_{1}\left(x\right)=C\left(a_{1},\ldots ,a_{K},b,c\right)\frac{{\left(1-\sum_{i=1}^{K}x_{i}\right)}^{b-1}\prod_{i=1}^{K}x_{i}^{a_{i}-1}}{{\left(1+\sum_{i=1}^{K}x_{i}\right)}^{c}} \end{array}$$

and

$$\begin{array}{c} f_{2}\left(x\right)=C\left(d_{1},\ldots ,d_{K},e,f\right)\frac{{\left(1-\sum_{i=1}^{K}x_{i}\right)}^{e-1}\prod_{i=1}^{K}x_{i}^{d_{i}-1}}{{\left(1+\sum_{i=1}^{K}x_{i}\right)}^{f}} \end{array}$$

for K≥2, $a_{1}>0,\ldots ,a_{K}>0$, b>0, −∞<c<∞, $d_{1}>0,\ldots ,d_{K}>0$, e>0, −∞<f<∞, $0\leq x_{1}\leq 1,\ldots ,0\leq x_{K}\leq 1$ and $x_{1}+\cdots +x_{K}\leq 1$. The corresponding KLD is

$$\begin{array}{cc} \; & KLD=\log\left[\frac{C\left(a_{1},\ldots ,a_{K},b,c\right)}{C\left(d_{1},\ldots ,d_{K},e,f\right)}\right]+\sum_{i=1}^{K}\left(a_{i}-d_{i}\right){\left.\frac{\partial }{\partial \alpha }\left[\frac{C\left(a_{1},\ldots ,a_{i},\ldots ,a_{K},b,c\right)}{C\left(a_{1},\ldots ,a_{i}+\alpha ,\ldots ,a_{K},b,c\right)}\right]\right|}_{\alpha =0}\\ \; & \;\;\;+\left(b-e\right){\left.\frac{\partial }{\partial \alpha }\left[\frac{C\left(a_{1},\ldots ,a_{i},\ldots ,a_{K},b,c\right)}{C\left(a_{1},\ldots ,a_{K},b+\alpha ,c\right)}\right]\right|}_{\alpha =0}\\ \; & \;\;\;-\left(c-f\right){\left.\frac{\partial }{\partial \alpha }\left[\frac{C\left(a_{1},\ldots ,a_{i},\ldots ,a_{K},b,c\right)}{C\left(a_{1},\ldots ,a_{K},b,c-\alpha \right)}\right]\right|}_{\alpha =0}. \end{array}$$

### 2.5. Multivariate Kotz Type Distribution [12]

Consider the joint probability density functions

$$\begin{array}{c} f_{1}\left(x_{1},\ldots ,x_{p}\right)=\frac{a\Gamma \left(\frac{p}{2}\right)q^{\frac{2N+p-2}{2a}}}{\pi^{\frac{p}{2}}\Gamma \left(\frac{2N+p-2}{2a}\right)}{\left|\Sigma_{1}\right|}^{-\frac{1}{2}}{\left(x^{T}\Sigma_{1}^{-1}x\right)}^{N-1}\exp\left\{-q{\left(x^{T}\Sigma_{1}^{-1}x\right)}^{a}\right\} \end{array}$$

and

$$\begin{array}{c} f_{2}\left(x_{1},\ldots ,x_{p}\right)=\frac{b\Gamma \left(\frac{p}{2}\right)s^{\frac{2M+p-2}{2b}}}{\pi^{\frac{p}{2}}\Gamma \left(\frac{2M+p-2}{2b}\right)}{\left|\Sigma_{2}\right|}^{-\frac{1}{2}}{\left(x^{T}\Sigma_{2}^{-1}x\right)}^{M-1}\exp\left\{-s{\left(x^{T}\Sigma_{2}^{-1}x\right)}^{b}\right\} \end{array}$$

for $-\infty <x_{1}<\infty ,\ldots ,-\infty <x_{p}<\infty$, a>0, q>0, $N>1-\frac{p}{2}$, b>0, s>0, $M>1-\frac{p}{2}$ and $\Sigma_{1}$, $\Sigma_{2}$ positive definite symmetric matrices. The corresponding KLD is

KLD=logaq2N+p−22aΓ2M+p−22bbs2M+p−22bΓ2N+p−22a+12logΣ2Σ1+N−1aψ2N+p−22a−logq−2N+p−22a−(M−1)Γp2aπp2ψp+2N−22a−logq∏j=1p−1Bp−j2,12−(M−1)Γp2πp2logλ1∏j=1p−1Bp−j2,12+(M−1)Γp2πp2∑k=1∞(−1)kk∑i1+⋯+ip−1=kki1,…,ip−1×∏j=1p−1λj+1−λjλ1ijB∑ℓ=jp−1iℓ+p−j2,12+sΓp2Γ2N+p+2b−22aπp2qbaΓ2N+p−22a∑r1=0∞∑r2=0r1⋯∑rp−1=0rp−2br1r1r2⋯rp−2rp−1λ1b−r1×∏j=1p−1λj+1−λjrj−rj+1Brj+p−j2,12

provided that the infinite sum converges.

### 2.6. Multivariate Logistic Distribution [6]

Consider the joint probability density functions

$$\begin{array}{c} f_{1}\left(x_{1},\ldots ,x_{p}\right)=\frac{p!a_{1}\cdots a_{p}\exp\left(-a_{1}x_{1}-\cdots -a_{p}x_{p}\right)}{{\left[1+\exp\left(-a_{1}x_{1}\right)+\cdots +\exp\left(-a_{p}x_{p}\right)\right]}^{p+1}} \end{array}$$

and

$$\begin{array}{c} f_{2}\left(x_{1},\ldots ,x_{p}\right)=\frac{p!b_{1}\cdots b_{p}\exp\left(-b_{1}x_{1}-\cdots -b_{p}x_{p}\right)}{{\left[1+\exp\left(-b_{1}x_{1}\right)+\cdots +\exp\left(-b_{p}x_{p}\right)\right]}^{p+1}} \end{array}$$

for $-\infty <x_{1}<\infty ,\ldots ,-\infty <x_{p}<\infty$, $a_{1}>0,\ldots ,a_{p}>0$ and $b_{1}>0,\ldots ,b_{p}>0$. The corresponding KLD is

KLD=loga1⋯apb1⋯bp−(p+1)∑k=1∞(−1)kk∑i1+⋯+ip=kki1,…,ip∏j=1pΓaj+ijbjajΓ1−∑j=1pijbjaj−(p+1)Γ′(p+1)−Γ(p+1)Γ′(1)p!

provided that $\sum_{j=1}^{p}\frac{i_{j}b_{j}}{a_{j}}<1$ and the infinite series converges.

### 2.7. Multivariate Logistic Distribution [7]

Consider the joint probability density functions

$$\begin{array}{c} f_{1}\left(x_{1},\ldots ,x_{p}\right)=\frac{{\left(b\right)}_{p}a_{1}\cdots a_{p}\exp\left(-a_{1}x_{1}-\cdots -a_{p}x_{p}\right)}{{\left[1+\exp\left(-a_{1}x_{1}\right)+\cdots +\exp\left(-a_{p}x_{p}\right)\right]}^{b+p}} \end{array}$$

and

$$\begin{array}{c} f_{2}\left(x_{1},\ldots ,x_{p}\right)=\frac{{\left(d\right)}_{p}c_{1}\cdots c_{p}\exp\left(-c_{1}x_{1}-\cdots -c_{p}x_{p}\right)}{{\left[1+\exp\left(-c_{1}x_{1}\right)+\cdots +\exp\left(-c_{p}x_{p}\right)\right]}^{d+p}} \end{array}$$

for $-\infty <x_{1}<\infty ,\ldots ,-\infty <x_{p}<\infty$, $a_{1}>0,\ldots ,a_{p}>0$, $c_{1}>0,\ldots ,c_{p}>0$, b>0 and d>0. The corresponding KLD is

KLD=log(b)pa1⋯ap(d)pb1⋯bp−d+pΓ(b)∑k=1∞(−1)kk∑i1+⋯+ip=kki1,…,ip∏j=1pΓaj+ijcjajΓb−∑j=1pijcjaj−(b+p)Γ(b)Γ′(b+p)−Γ(b+p)Γ′(b)Γ(b)Γ(b+p)

provided that $\sum_{j=1}^{p}\frac{i_{j}c_{j}}{a_{j}}<b$ and the infinite series converges.

### 2.8. Sarabia [8]’s Multivariate Normal Distribution

Consider the joint probability density functions

$$\begin{array}{c} f_{1}\left(x_{1},\ldots ,x_{p}\right)=\frac{\sqrt{a_{1}\cdots a_{p}}\beta_{p}\left(c,a_{1},\ldots ,a_{p}\right)}{{\left(2\pi \right)}^{\frac{p}{2}}}\exp\left\{-\frac{1}{2}\left[\sum_{i=1}^{p}\left(a_{i}x_{i}^{2}\right)+c\prod_{i=1}^{p}\left(a_{i}x_{i}^{2}\right)\right]\right\} \end{array}$$

and

$$\begin{array}{c} f_{2}\left(x_{1},\ldots ,x_{p}\right)=\frac{\sqrt{b_{1}\cdots b_{p}}\beta_{p}\left(d,b_{1},\ldots ,b_{p}\right)}{{\left(2\pi \right)}^{\frac{p}{2}}}\exp\left\{-\frac{1}{2}\left[\sum_{i=1}^{p}\left(b_{i}x_{i}^{2}\right)+d\prod_{i=1}^{p}\left(b_{i}x_{i}^{2}\right)\right]\right\} \end{array}$$

for $-\infty <x_{1}<\infty ,\ldots ,-\infty <x_{p}<\infty$, $a_{1}>0,\ldots ,a_{p}>0$, $b_{1}>0,\ldots ,b_{p}>0$, c>0 and d>0, where $\beta_{p}\left(c,a_{1},\ldots ,a_{p}\right)$ and $\beta_{p}\left(d,b_{1},\ldots ,b_{p}\right)$ denote normalizing constants. The corresponding KLD is

$$\begin{array}{cc} KLD & =\log\left[\frac{\sqrt{a_{1}\cdots a_{p}}\beta_{p}\left(c,a_{1},\ldots ,a_{p}\right)}{\sqrt{b_{1}\cdots b_{p}}\beta_{p}\left(d,b_{1},\ldots ,b_{p}\right)}\right]\\ \; & \;+\left[\frac{1}{2}-\frac{c\beta_{p}^{\prime }\left(c,a_{1},\ldots ,a_{p}\right)}{\beta_{p}\left(c,a_{1},\ldots ,a_{p}\right)}\right]\sum_{i=1}^{p}\left(\frac{b_{i}}{a_{i}}-1\right)\\ \; & \;+\left(\frac{db_{1}\cdots b_{p}}{a_{1}\cdots a_{p}}-c\right)\frac{\beta_{p}^{\prime }\left(c,a_{1},\ldots ,a_{p}\right)}{\beta_{p}\left(c,a_{1},\ldots ,a_{p}\right)}, \end{array}$$

where $\beta_{p}^{\prime }\left(c,a_{1},\ldots ,a_{p}\right)=\frac{\partial }{\partial c}\beta_{p}\left(c,a_{1},\ldots ,a_{p}\right)$.

### 2.9. Multivariate Pearson Type II Distribution

Consider the joint probability density functions

$$\begin{array}{c} f_{1}\left(x\right)=\frac{\pi^{-\frac{K}{2}}\Gamma \left(\frac{K}{2}\right)\Gamma \left(\frac{K}{2}+a+b-1\right)}{\Gamma \left(a\right)\Gamma \left(\frac{K}{2}+b-1\right)}{\left(\sum_{i=1}^{K}x_{i}^{2}\right)}^{b-1}{\left(1-\sum_{i=1}^{K}x_{i}^{2}\right)}^{a-1} \end{array}$$

and

$$\begin{array}{c} f_{2}\left(x\right)=\frac{\pi^{-\frac{K}{2}}\Gamma \left(\frac{K}{2}\right)\Gamma \left(\frac{K}{2}+c+d-1\right)}{\Gamma \left(c\right)\Gamma \left(\frac{K}{2}+d-1\right)}{\left(\sum_{i=1}^{K}x_{i}^{2}\right)}^{d-1}{\left(1-\sum_{i=1}^{K}x_{i}^{2}\right)}^{c-1} \end{array}$$

for K≥2, a>0, b>0, c>0, d>0 and $0<x_{1}^{2}+\cdots +x_{K}^{2}<1$. The corresponding KLD is

$$\begin{array}{cc} \; & KLD=\log\left[\frac{\Gamma \left(\frac{K}{2}+a+b-1\right)\Gamma \left(c\right)\Gamma \left(\frac{K}{2}+d-1\right)}{\Gamma \left(a\right)\Gamma \left(\frac{K}{2}+b-1\right)\Gamma \left(\frac{K}{2}+c+d-1\right)}\right]\\ \; & \;\;\;+\left(b-d\right)\left[\psi \left(\frac{K}{2}+b-1\right)-\psi \left(\frac{K}{2}+a+b-1\right)\right]\\ \; & \;\;\;+\left(a-c\right)\left[\psi \left(a\right)-\psi \left(\frac{K}{2}+a+b-1\right)\right]. \end{array}$$

### 2.10. Multivariate Selberg Beta Distribution [13]

Consider the joint probability density functions

$$\begin{array}{c} f_{1}\left(x\right)=C\left(a,b,c\right){\left|\prod_{1\leq i<j\leq p}\left(x_{i}-x_{j}\right)\right|}^{2c}\prod_{i=1}^{p}\left[x_{i}^{a-1}{\left(1-x_{i}\right)}^{b-1}\right] \end{array}$$

and

$$\begin{array}{c} f_{2}\left(x\right)=C\left(d,e,f\right){\left|\prod_{1\leq i<j\leq p}\left(x_{i}-x_{j}\right)\right|}^{2f}\prod_{i=1}^{p}\left[x_{i}^{d-1}{\left(1-x_{i}\right)}^{e-1}\right] \end{array}$$

for a>0, b>0, c>0, d>0, e>0, f>0 and $0<x_{1}<1,\ldots ,0<x_{p}<1$. The corresponding KLD is

$$\begin{array}{cc} \; & KLD=\log\left[\frac{C\left(d,e,f\right)}{C\left(a,b,c\right)}\right]+\left(a-d\right){\left.\frac{\partial }{\partial \alpha }\left[\frac{C(a,b,c)}{C(a+\alpha ,b,c)}\right]\right|}_{\alpha =0}\\ \; & \;\;\;+\left(b-e\right){\left.\frac{\partial }{\partial \alpha }\left[\frac{C(a,b,c)}{C(a,b+\alpha ,c)}\right]\right|}_{\alpha =0}\\ \; & \;\;\;+2\left(c-f\right){\left.\frac{\partial }{\partial \alpha }\left[\frac{C(a,b,c)}{C(a,b,c+\alpha )}\right]\right|}_{\alpha =0}. \end{array}$$

### 2.11. Multivariate Weighted Exponential Distribution [14]

Consider the joint probability density functions

$$\begin{array}{c} f_{1}\left(x_{1},\ldots ,x_{p}\right)=\frac{\left(\sum_{i=1}^{p}a_{i}\right)\left(\prod_{i=1}^{p}a_{i}\right)}{a_{p+1}}\left\{1-\exp\left[-a_{p+1}\min\left(x_{1},\ldots ,x_{p}\right)\right]\right\}\left[\prod_{i=1}^{p}\exp\left(-a_{i}x_{i}\right)\right] \end{array}$$

and

$$\begin{array}{c} f_{2}\left(x_{1},\ldots ,x_{p}\right)=\frac{\left(\sum_{i=1}^{p}b_{i}\right)\left(\prod_{i=1}^{p}b_{i}\right)}{b_{p+1}}\left\{1-\exp\left[-b_{p+1}\min\left(x_{1},\ldots ,x_{p}\right)\right]\right\}\left[\prod_{i=1}^{p}\exp\left(-b_{i}x_{i}\right)\right] \end{array}$$

for $x_{1}>0,\ldots ,x_{p}>0$, $a_{1}>0,\ldots ,a_{p+1}>0$ and $b_{1}>0,\ldots ,b_{p+1}>0$. The corresponding KLD is

$$\begin{array}{cc} KLD & =\log\left[\frac{b_{p+1}\left(\sum_{i=1}^{p}a_{i}\right)\left(\prod_{i=1}^{p}a_{i}\right)}{a_{p+1}\left(\sum_{i=1}^{p}b_{i}\right)\left(\prod_{i=1}^{p}b_{i}\right)}\right]+\sum_{i=1}^{p}\left(b_{i}-a_{i}\right)\left(\frac{1}{a_{i}}+\frac{1}{a_{p+1}}\right)\\ \; & \;\;\;-\sum_{k=1}^{\infty }\frac{\left(a_{1}+\cdots +a_{p}\right)\left(a_{1}+\cdots +a_{p}+a_{p+1}\right)}{k\left(a_{1}+\cdots +a_{p}+ka_{p+1}\right)\left[a_{1}+\cdots +a_{p}+\left(k+1\right)a_{p+1}\right]}\\ \; & \;\;\;+\sum_{k=1}^{\infty }\frac{\left(a_{1}+\cdots +a_{p}\right)\left(a_{1}+\cdots +a_{p}+a_{p+1}\right)}{k\left(a_{1}+\cdots +a_{p}+kb_{p+1}\right)\left[a_{1}+\cdots +a_{p}+a_{p+1}+kb_{p+1}\right]}, \end{array}$$

which follows from properties stated in [14] provided that the infinite series converge.

### 2.12. Von Mises Distribution

Consider the joint probability density functions

$$\begin{array}{c} f_{1}\left(x\right)=\frac{\kappa_{1}^{\frac{p}{2}-1}\exp\left(\kappa_{1}\mu_{1}^{T}x\right)}{{\left(2\pi \right)}^{\frac{p}{2}}I_{\frac{p}{2}-1}\left(\kappa_{1}\right)} \end{array}$$

and

$$\begin{array}{c} f_{2}\left(x\right)=\frac{\kappa_{2}^{\frac{p}{2}-1}\exp\left(\kappa_{2}\mu_{2}^{T}x\right)}{{\left(2\pi \right)}^{\frac{p}{2}}I_{\frac{p}{2}-1}\left(\kappa_{2}\right)} \end{array}$$

for $\kappa_{1}>0$, $\kappa_{2}>0$, $\mu_{1}^{T}\mu_{1}=1$, $\mu_{2}^{T}\mu_{2}=1$ and $x^{T}x=1$. The corresponding KLD is

$$\begin{array}{c} KLD=\log\left[\frac{\kappa_{1}^{\frac{p}{2}-1}}{\kappa_{2}^{\frac{p}{2}-1}}\frac{I_{\frac{p}{2}-1}\left(\kappa_{2}\right)}{I_{\frac{p}{2}-1}\left(\kappa_{1}\right)}\right]+\left(\kappa_{1}-\kappa_{2}\mu_{2}^{T}\mu_{1}\right). \end{array}$$

## 3. Exact Expressions for Matrix-Variate Distributions

In this section, we state exact expressions for (1) for matrix-variate beta, matrix-variate Dirichlet, matrix-variate gamma, matrix-variate Gauss hypergeometric, matrix-variate inverse beta, matrix-variate inverse gamma, matrix-variate Kummer beta, matrix-variate Kummer gamma, matrix-variate normal and matrix-variate two-sided power distributions.

### 3.1. Matrix-Variate Beta Distribution [15]

Consider the joint probability density functions

$$\begin{array}{c} f_{1}\left(x\right)=\frac{{\left|\Omega -x\right|}^{a-\frac{p+1}{2}}{\left|x\right|}^{b-\frac{p+1}{2}}}{{\left|\Omega \right|}^{a+b}B_{p}\left(a,b\right)} \end{array}$$

and

$$\begin{array}{c} f_{2}\left(x\right)=\frac{{\left|\Omega -x\right|}^{c-\frac{p+1}{2}}{\left|x\right|}^{d-\frac{p+1}{2}}}{{\left|\Omega \right|}^{c+d}B_{p}\left(c,d\right)} \end{array}$$

for $a>\frac{p-1}{2}$, $b>\frac{p-1}{2}$, $c>\frac{p-1}{2}$, $d>\frac{p-1}{2}$ and Ω, x, Ω−x being p×p positive definite matrices. The corresponding KLD is

$$\begin{array}{cc} \; & KLD=\log\left[\frac{{\left|\Omega \right|}^{c+d}B_{p}\left(c,d\right)}{{\left|\Omega \right|}^{a+b}B_{p}\left(a,b\right)}\right]+\left(a-c\right){\left.\frac{\partial }{\partial \alpha }\left\{\frac{{\left|\Omega \right|}^{\alpha }B_{p}\left(\alpha +a,b\right)}{B_{p}\left(a,b\right)}\right\}\right|}_{\alpha =0}\\ \; & \;\;\;+\left(b-d\right){\left.\frac{\partial }{\partial \alpha }\left\{\frac{{\left|\Omega \right|}^{\alpha }B_{p}\left(a,\alpha +b\right)}{B_{p}\left(a,b\right)}\right\}\right|}_{\alpha =0}. \end{array}$$

### 3.2. Matrix-Variate Dirichlet Distribution

Consider the joint probability density functions

$$\begin{array}{c} f_{1}\left(x_{1},\ldots ,x_{n}\right)=\frac{1}{B_{p}\left(a_{1},\ldots ,a_{n};a_{n+1}\right)}{\left|x_{1}\right|}^{a_{1}-p}\cdots {\left|x_{n}\right|}^{a_{n}-p}{\left|I_{p}-\sum_{i=1}^{n}x_{i}\right|}^{a_{n+1}-p} \end{array}$$

and

$$\begin{array}{c} f_{2}\left(x_{1},\ldots ,x_{n}\right)=\frac{1}{B_{p}\left(b_{1},\ldots ,b_{n};b_{n+1}\right)}{\left|x_{1}\right|}^{b_{1}-p}\cdots {\left|x_{n}\right|}^{b_{n}-p}{\left|I_{p}-\sum_{i=1}^{n}x_{i}\right|}^{b_{n+1}-p} \end{array}$$

for $a_{1}>\frac{p-1}{2},\ldots ,a_{n+1}>\frac{p-1}{2}$, $b_{1}>\frac{p-1}{2},\ldots ,b_{n+1}>\frac{p-1}{2}$ and $x_{1},\ldots ,x_{n}$, $I_{p}-x_{1}-\cdots -x_{n}$ being p×p positive definite matrices. The corresponding KLD is

$$\begin{array}{cc} \; & KLD=\log\left[\frac{B_{p}\left(b_{1},\ldots ,b_{n};b_{n+1}\right)}{B_{p}\left(a_{1},\ldots ,a_{n};a_{n+1}\right)}\right]+\left(a_{n+1}-b_{n+1}\right){\left.\frac{\partial }{\partial \alpha }\frac{B_{p}\left(a_{1},\ldots ,a_{n};a_{n+1}+\alpha \right)}{B_{p}\left(a_{1},\ldots ,a_{n};a_{n+1}\right)}\right|}_{\alpha =0}\\ \; & \;\;\;+\sum_{i=1}^{n}\left(a_{i}-b_{i}\right){\left.\frac{\partial }{\partial \alpha }\frac{B_{p}\left(a_{1},\ldots ,a_{i}+\alpha ,\ldots ,a_{n};a_{n+1}\right)}{B_{p}\left(a_{1},\ldots ,a_{i},\ldots ,a_{n};a_{n+1}\right)}\right|}_{\alpha =0}. \end{array}$$

### 3.3. Matrix-Variate Gamma Distribution

Consider the joint probability density functions

$$\begin{array}{c} f_{1}\left(x\right)=\frac{{\left|\Sigma_{1}\right|}^{-a}}{b^{ap}\Gamma_{p}\left(a\right)}{\left|x\right|}^{a-\frac{p+1}{2}}\exp\left[\mathrm{tr}\;\left(-\frac{1}{b}\Sigma_{1}^{-1}x\right)\right] \end{array}$$

and

$$\begin{array}{c} f_{2}\left(x\right)=\frac{{\left|\Sigma_{2}\right|}^{-c}}{d^{cp}\Gamma_{p}\left(c\right)}{\left|x\right|}^{c-\frac{p+1}{2}}\exp\left[\mathrm{tr}\;\left(-\frac{1}{d}\Sigma_{2}^{-1}x\right)\right] \end{array}$$

for $a>\frac{p-1}{2}$, b>0, $c>\frac{p-1}{2}$, d>0 and x, $\Sigma_{1}$, $\Sigma_{2}$ being p×p positive definite symmetric matrices. The corresponding KLD is

$$\begin{array}{c} KLD=\log\left[\frac{d^{pc}\Gamma_{p}\left(c\right)}{b^{pa}\Gamma_{p}\left(a\right)}\frac{{\left|\Sigma_{2}\right|}^{c}}{{\left|\Sigma_{1}\right|}^{a}}\right]+\frac{a-c}{\Gamma_{p}\left(a\right)}{\left.\frac{\partial }{\partial \alpha }\left[{\left|\Sigma_{1}\right|}^{\alpha }b^{p\alpha }\Gamma_{p}\left(a+\alpha \right)\right]\right|}_{\alpha =0}+\frac{2ap}{b}-\mathrm{tr}\;\left[-\frac{2a}{d}\Sigma_{2}^{-1}\Sigma_{1}\right]. \end{array}$$

### 3.4. Matrix-Variate Gauss Hypergeometric Distribution [16]

Consider the joint probability density functions

$$\begin{array}{c} f_{1}\left(x\right)=\frac{{\left|x\right|}^{a-\frac{p+1}{2}}{\left|I_{p}-x\right|}^{b-\frac{p+1}{2}}}{B_{p}\left(a,b\right){\;}_{2}F_{1}\left(a,c;a+b;-B\right){\left|I_{p}+Bx\right|}^{c}} \end{array}$$

and

$$\begin{array}{c} f_{2}\left(x\right)=\frac{{\left|x\right|}^{d-\frac{p+1}{2}}{\left|I_{p}-x\right|}^{e-\frac{p+1}{2}}}{B_{p}\left(d,e\right){\;}_{2}F_{1}\left(d,f;d+e;-B\right){\left|I_{p}+Bx\right|}^{f}} \end{array}$$

for $a>\frac{p-1}{2}$, $b>\frac{p-1}{2}$, 0≤c<∞, $d>\frac{p-1}{2}$, $e>\frac{p-1}{2}$, 0≤f<∞ and x, $I_{p}-x$, B, $I_{p}+B$ being p×p positive definite matrices, where $\Gamma_{p}\left(c\right)$ and $\Gamma_{p}\left(f\right)$ are assumed to exist. The corresponding KLD is

$$\begin{array}{cc} \; & KLD=\log\left[\frac{B_{p}\left(d,e\right){\;}_{2}F_{1}\left(d,f;d+e;-B\right)}{B_{p}\left(a,b\right){\;}_{2}F_{1}\left(a,c;a+b;-B\right)}\right]\\ \; & \;\;\;+\left(a-d\right){\left.\frac{\partial }{\partial \alpha }\frac{B_{p}\left(a+\alpha ,b\right){\;}_{2}F_{1}\left(a+\alpha ,c;a+b+\alpha ;-B\right)}{B_{p}\left(a,b\right){\;}_{2}F_{1}\left(a,c;a+b;-B\right)}\right|}_{\alpha =0}\\ \; & \;\;\;+\left(b-e\right){\left.\frac{\partial }{\partial \alpha }\frac{B_{p}\left(a,b+\alpha \right){\;}_{2}F_{1}\left(a,c;a+b+\alpha ;-B\right)}{B_{p}\left(a,b\right){\;}_{2}F_{1}\left(a,c;a+b;-B\right)}\right|}_{\alpha =0}\\ \; & \;\;\;+\left(f-c\right){\left.\frac{\partial }{\partial \alpha }\frac{{\;}_{2}F_{1}\left(a,c-\alpha ;a+b;-B\right)}{{\;}_{2}F_{1}\left(a,c;a+b;-B\right)}\right|}_{\alpha =0}. \end{array}$$

### 3.5. Matrix-Variate Inverse Beta Distribution

Consider the joint probability density functions

$$\begin{array}{c} f_{1}\left(x\right)=\frac{{\left|\Omega +x\right|}^{-a-b}{\left|x\right|}^{b-\frac{p+1}{2}}}{{\left|\Omega \right|}^{-a}B_{p}\left(a,b\right)} \end{array}$$

and

$$\begin{array}{c} f_{2}\left(x\right)=\frac{{\left|\Omega +x\right|}^{-c-d}{\left|x\right|}^{d-\frac{p+1}{2}}}{{\left|\Omega \right|}^{-c}B_{p}\left(c,d\right)} \end{array}$$

for $a>\frac{p-1}{2}$, $b>\frac{p-1}{2}$, $c>\frac{p-1}{2}$, $d>\frac{p-1}{2}$ and x, Ω, Ω+x being p×p positive definite matrices. The corresponding KLD is

$$\begin{array}{cc} \; & KLD=\log\left[\frac{{\left|\Omega \right|}^{a-c}B_{p}\left(c,d\right)}{B_{p}\left(a,b\right)}\right]+\left(c+d-a-b\right){\left.\frac{\partial }{\partial \alpha }\left\{\frac{{\left|\Omega \right|}^{\alpha }B_{p}\left(a-\alpha ,b\right)}{B_{p}\left(a,b\right)}\right\}\right|}_{\alpha =0}\\ \; & \;\;\;+\left(b-d\right){\left.\frac{\partial }{\partial \alpha }\left\{\frac{{\left|\Omega \right|}^{\alpha }B_{p}\left(a-\alpha ,\alpha +b\right)}{B_{p}\left(a,b\right)}\right\}\right|}_{\alpha =0}. \end{array}$$

### 3.6. Matrix-Variate Inverse Gamma Distribution

Consider the joint probability density functions

$$\begin{array}{c} f_{1}\left(x\right)=\frac{{\left|\Sigma_{1}\right|}^{a}}{b^{ap}\Gamma_{p}\left(a\right)}{\left|x\right|}^{-a-\frac{p+1}{2}}\exp\left[\mathrm{tr}\;\left(-\frac{1}{b}\Sigma_{1}x^{-1}\right)\right] \end{array}$$

and

$$\begin{array}{c} f_{2}\left(x\right)=\frac{{\left|\Sigma_{2}\right|}^{c}}{d^{cp}\Gamma_{p}\left(c\right)}{\left|x\right|}^{-c-\frac{p+1}{2}}\exp\left[\mathrm{tr}\;\left(-\frac{1}{d}\Sigma_{2}x^{-1}\right)\right] \end{array}$$

for $a>\frac{p-1}{2}$, b>0, $c>\frac{p-1}{2}$, d>0 and x, $\Sigma_{1}$, $\Sigma_{2}$ being p×p positive definite symmetric matrices. The corresponding KLD is

$$\begin{array}{cc} \; & KLD=\log\left[\frac{d^{pc}\Gamma_{p}\left(c\right)}{b^{pa}\Gamma_{p}\left(a\right)}\frac{{\left|\Sigma_{1}\right|}^{a}}{{\left|\Sigma_{2}\right|}^{c}}\right]+\frac{c-a}{\Gamma_{p}\left(a\right)}{\left.\frac{\partial }{\partial \alpha }\left[\frac{{\left|\Sigma_{1}\right|}^{\alpha }\Gamma_{p}\left(a-\alpha \right)}{b^{p\alpha }}\right]\right|}_{\alpha =0}\\ \; & \;\;\;+\frac{2a}{d}\;\mathrm{tr}\;\left[\Sigma_{2}\Sigma_{1}^{-1}\right]-\frac{2ap}{b}. \end{array}$$

### 3.7. Matrix-Variate Kummer Beta Distribution [17]

Consider the joint probability density functions

$$\begin{array}{c} f_{1}\left(x\right)=\frac{{\left|x\right|}^{a-\frac{p+1}{2}}{\left|I_{p}-x\right|}^{b-\frac{p+1}{2}}\exp\left[-\mathrm{tr}\;\left(B_{1}x\right)\right]}{B_{p}\left(a,b\right){\;}_{1}F_{1}\left(a,a+b;-B_{1}\right)} \end{array}$$

and

$$\begin{array}{c} f_{2}\left(x\right)=\frac{{\left|x\right|}^{c-\frac{p+1}{2}}{\left|I_{p}-x\right|}^{d-\frac{p+1}{2}}\exp\left[-\mathrm{tr}\;\left(B_{2}x\right)\right]}{B_{p}\left(c,d\right){\;}_{1}F_{1}\left(c;c+d;-B_{2}\right)} \end{array}$$

for $a>\frac{p-1}{2}$, $b>\frac{p-1}{2}$, $c>\frac{p-1}{2}$, $d>\frac{p-1}{2}$ and x, $I_{p}-x$, $B_{1}$, $B_{2}$ being p×p positive definite matrices. The corresponding KLD is

$$\begin{array}{cc} \; & KLD=\log\left[\frac{B_{p}\left(c,d\right){\;}_{1}F_{1}\left(c;c+d;-B_{2}\right)}{B_{p}\left(a,b\right){\;}_{1}F_{1}\left(a,a+b;-B_{1}\right)}\right]\\ \; & \;\;\;+\left(a-c\right){\left.\frac{\partial }{\partial \alpha }\frac{B_{p}\left(a+\alpha ,b\right){\;}_{1}F_{1}\left(a+\alpha ;a+b+\alpha ;-B_{1}\right)}{B_{p}\left(a,b\right){\;}_{1}F_{1}\left(a;a+b;-B_{1}\right)}\right|}_{\alpha =0}\\ \; & \;\;\;+\left(b-d\right){\left.\frac{\partial }{\partial \alpha }\frac{B_{p}\left(a,b+\alpha \right){\;}_{1}F_{1}\left(a;a+b+\alpha ;-B_{1}\right)}{B_{p}\left(a,b\right){\;}_{1}F_{1}\left(a;a+b;-B_{1}\right)}\right|}_{\alpha =0}\\ \; & \;\;\;+\mathrm{tr}\;\left[\left(B_{2}-B_{1}\right){\left.\frac{\partial }{\partial z}\frac{{\;}_{1}F_{1}\left(a;a+b;z-B_{1}\right)}{{\;}_{1}F_{1}\left(a;a+b;-B_{1}\right)}\right|}_{z=0}\right]. \end{array}$$

### 3.8. Matrix-Variate Kummer Gamma Distribution [18]

Consider the joint probability density functions

$$\begin{array}{c} f_{1}\left(x\right)=\frac{{\left|x\right|}^{a-\frac{p+1}{2}}{\left|I_{p}+x\right|}^{-b}\exp\left[-\mathrm{tr}\;\left(B_{1}x\right)\right]}{\Gamma_{p}\left(a\right)\;\Psi_{1}\left(a;a-b+\frac{p+1}{2};B_{1}\right)} \end{array}$$

and

$$\begin{array}{c} f_{2}\left(x\right)=\frac{{\left|x\right|}^{c-\frac{p+1}{2}}{\left|I_{p}+x\right|}^{-d}\exp\left[-\mathrm{tr}\;\left(B_{2}x\right)\right]}{\Gamma_{p}\left(c\right)\;\Psi_{1}\left(c;c-d+\frac{p+1}{2};B_{2}\right)} \end{array}$$

for $a>\frac{p-1}{2}$, −∞<b<∞, $c>\frac{p-1}{2}$, −∞<d<∞ and x, $I_{p}+x$, $B_{1}$, $B_{2}$ being p×p positive definite matrices, where $\Psi_{1}\left(a;a-b+\frac{p+1}{2};B_{1}\right)$ and $\Psi_{1}\left(c;c-d+\frac{p+1}{2};B_{2}\right)$ denote Kummer functions with matrix arguments and the parameters are chosen such that these functions exist. The corresponding KLD is

$$\begin{array}{cc} \; & KLD=\log\left[\frac{\Gamma_{p}\left(c\right)\;\Psi_{1}\left(c;c-d+\frac{p+1}{2};B_{2}\right)}{\Gamma_{p}\left(a\right)\;\Psi_{1}\left(a;a-b+\frac{p+1}{2};B_{1}\right)}\right]\\ \; & \;\;\;+\left(a-c\right){\left.\frac{\partial }{\partial \alpha }\frac{\Gamma_{p}\left(a+\alpha \right)\Psi_{1}\left(a+\alpha ;a-b+\alpha +\frac{p+1}{2};B_{1}\right)}{\Gamma_{p}\left(a\right)\Psi_{1}\left(a;a-b+\frac{p+1}{2};B_{1}\right)}\right|}_{\alpha =0}\\ \; & \;\;\;+\left(d-p\right){\left.\frac{\partial }{\partial \alpha }\frac{\Psi_{1}\left(a;a-b+\alpha +\frac{p+1}{2};B_{1}\right)}{\Psi_{1}\left(a;a-b+\frac{p+1}{2};B_{1}\right)}\right|}_{\alpha =0}\\ \; & \;\;\;+\mathrm{tr}\;\left[\left(B_{2}-B_{1}\right){\left.\frac{\partial }{\partial z}\frac{\Psi_{1}\left(a;a-b+\frac{p+1}{2};B_{1}-z\right)}{\Psi_{1}\left(a;a-b+\frac{p+1}{2};B_{1}\right)}\right|}_{z=0}\right]. \end{array}$$

### 3.9. Matrix-Variate Normal Distribution

Consider the joint probability density functions

$$\begin{array}{c} f_{1}\left(x\right)=\frac{1}{{\left(2\pi \right)}^{\frac{np}{2}}{\left|V_{1}\right|}^{\frac{n}{2}}{\left|U_{1}\right|}^{\frac{p}{2}}}\exp\left\{-\frac{1}{2}\;\mathrm{tr}\;\left[V_{1}^{-1}{\left(x-M_{1}\right)}^{T}U_{1}^{-1}\left(x-M_{1}\right)\right]\right\} \end{array}$$

and

$$\begin{array}{c} f_{2}\left(x\right)=\frac{1}{{\left(2\pi \right)}^{\frac{np}{2}}{\left|V_{2}\right|}^{\frac{n}{2}}{\left|U_{2}\right|}^{\frac{p}{2}}}\exp\left\{-\frac{1}{2}\;\mathrm{tr}\;\left[V_{2}^{-1}{\left(x-M_{2}\right)}^{T}U_{2}^{-1}\left(x-M_{2}\right)\right]\right\} \end{array}$$

for $U_{1}$, $U_{2}$ being positive definite symmetric matrices of dimension n×n, $V_{1}$, $V_{2}$ being positive definite symmetric matrices of dimension p×p and $M_{1}$, $M_{2}$ being matrices of dimension n×p. The corresponding KLD is

$$\begin{array}{cc} \; & KLD=\log\left[\frac{{\left|V_{2}\right|}^{\frac{n}{2}}{\left|U_{2}\right|}^{\frac{p}{2}}}{{\left|V_{1}\right|}^{\frac{n}{2}}{\left|U_{1}\right|}^{\frac{p}{2}}}\right]+\frac{1}{2}\mathrm{tr}\;\left(U_{1}\right)\;\mathrm{tr}\;\left(V_{2}^{-1}V_{1}U_{2}^{-1}\right)+\frac{1}{2}\;\mathrm{tr}\;\left(V_{2}^{-1}M_{1}^{T}M_{1}U_{2}^{-1}\right)\\ \; & \;\;\;-\frac{1}{2}\;\mathrm{tr}\;\left(V_{2}^{-1}M_{1}^{T}M_{2}U_{2}^{-1}\right)-\frac{1}{2}\;\mathrm{tr}\;\left(V_{2}^{-1}M_{2}^{T}M_{1}U_{2}^{-1}\right)\\ \; & \;\;\;+\frac{1}{2}\;\mathrm{tr}\;\left(V_{2}^{-1}M_{2}^{T}M_{2}U_{2}^{-1}\right)-\frac{1}{2}\;\mathrm{tr}\;\left(U_{1}\right)\;\mathrm{tr}\;\left(U_{1}^{-1}\right). \end{array}$$

### 3.10. Matrix-Variate Two-Sided Power Distribution [19]

Consider the joint probability density functions

$$\begin{array}{c} f_{1}\left(x\right)=C\left(a\right)\left\{\begin{array}{cc} {\left|x\right|}^{a-\frac{p+1}{2}}{\left|B\right|}^{-a+\frac{p+1}{2}}, & 0_{p}<x\leq B,\\ {\left|I_{p}-x\right|}^{a-\frac{p+1}{2}}{\left|I_{p}-B\right|}^{-a+\frac{p+1}{2}},\; & B\leq x\leq I_{p} \end{array}\right. \end{array}$$

and

$$\begin{array}{c} f_{2}\left(x\right)=C\left(b\right)\left\{\begin{array}{cc} {\left|x\right|}^{b-\frac{p+1}{2}}{\left|B\right|}^{-b+\frac{p+1}{2}}, & 0_{p}<x\leq B,\\ {\left|I_{p}-x\right|}^{b-\frac{p+1}{2}}{\left|I_{p}-B\right|}^{-b+\frac{p+1}{2}},\; & B\leq x\leq I_{p}, \end{array}\right. \end{array}$$

for $a>\frac{p-1}{2}$, $b>\frac{p-1}{2}$ and x, $I_{p}-x$, B, $I_{p}-B$ being p×p positive definite matrices, where

$$\begin{array}{c} C\left(a\right)={\left|B\right|}^{\frac{p+1}{2}}B_{p}\left(a,\frac{p+1}{2}\right)+{\left|I_{p}-B\right|}^{\frac{p+1}{2}}B_{p}\left(\frac{p+1}{2},a\right) \end{array}$$

and

$$\begin{array}{c} C\left(b\right)={\left|B\right|}^{\frac{p+1}{2}}B_{p}\left(b,\frac{p+1}{2}\right)+{\left|I_{p}-B\right|}^{\frac{p+1}{2}}B_{p}\left(\frac{p+1}{2},b\right). \end{array}$$

The corresponding KLD is

$$\begin{array}{cc} \; & KLD=\log\left[\frac{C(a)}{C(b)}\right]+\left(a-b\right){\left.\frac{\partial }{\partial \alpha }\left\{{\left|B\right|}^{\alpha +\frac{p+1}{2}}B_{p}\left(a+\alpha ,\frac{p+1}{2}\right)\right\}\right|}_{\alpha =0}\\ \; & \;\;\;+\left(a-b\right){\left.\frac{\partial }{\partial \alpha }\left\{{\left|I_{p}-B\right|}^{\alpha +\frac{p+1}{2}}B_{p}\left(\frac{p+1}{2},a+\alpha \right)\right\}\right|}_{\alpha =0}\\ \; & \;\;\;+\left(b-a\right){\left|B\right|}^{\frac{p+1}{2}}\log\left|B\right|B_{p}\left(a,\frac{p+1}{2}\right)\\ \; & \;\;\;+\left(b-a\right){\left|I_{p}-B\right|}^{\frac{p+1}{2}}\log\left|I_{p}-B\right|B_{p}\left(\frac{p+1}{2},a\right). \end{array}$$

## 4. Proofs

Before presenting the proofs of the expressions in Section 2 and Section 3, we state a lemma and give its proof.

### 4.1. A Technical Lemma

**Lemma 1.**  *Let*

$$\begin{array}{c} I\left(a_{1},\ldots ,a_{p},t_{1},\ldots ,t_{p},b\right)=\int_{R^{p}}\frac{\exp\left(-\sum_{j=1}^{p}t_{j}x_{j}\right)}{{\left[1+\sum_{j=1}^{p}\exp\left(-a_{j}x_{j}\right)\right]}^{b}}dx_{1}\cdots dx_{p} \end{array}$$

*for $t_{j}>0$, $a_{j}>0$, j=1,2,…,p and b>0. Then,*

$$\begin{array}{c} I\left(a_{1},\ldots ,a_{p},t_{1},\ldots ,t_{p},b\right)=\frac{1}{a_{1}\cdots a_{p}\Gamma \left(b\right)}\left[\prod_{j=1}^{p}\Gamma \left(\frac{t_{j}}{a_{j}}\right)\right]\Gamma \left(b-\sum_{j=1}^{p}\frac{t_{j}}{a_{j}}\right) \end{array}$$

*provided that $b-\sum_{j=1}^{p}\frac{t_{j}}{a_{j}}>0$.*

**Proof.**  Setting $y_{j}=\exp\left(-a_{j}x_{j}\right)$ and assuming the conditions in the lemma, we can write

$$\begin{array}{cc} \; & I\left(a_{1},\ldots ,a_{p},t_{1},\ldots ,t_{p},b\right)\\ \; & =\frac{1}{\Gamma (b)}\int_{R^{p}}\int_{0}^{\infty }t^{b-1}\exp\left\{-\left[1+\sum_{j=1}^{p}\exp\left(-a_{j}x_{j}\right)\right]t\right\}\exp\left(-\sum_{j=1}^{p}t_{j}x_{j}\right)dtdx_{1}\cdots dx_{p}\\ \; & =\frac{1}{\Gamma (b)}\int_{0}^{\infty }t^{b-1}e^{-t}\prod_{j=1}^{p}\left\{\int_{-\infty }^{\infty }\exp\left[-t_{j}x_{j}-t\exp\left(-a_{j}x_{j}\right)\right]dx_{j}\right\}dt\\ \; & =\frac{1}{a_{1}\cdots a_{p}\Gamma \left(b\right)}\int_{0}^{\infty }t^{b-1}e^{-t}\prod_{j=1}^{p}\left[\int_{0}^{\infty }y_{j}^{\frac{t_{j}}{a_{j}}-1}\exp\left(-ty_{j}\right)dy_{j}\right]dt\\ \; & =\frac{1}{a_{1}\cdots a_{p}\Gamma \left(b\right)}\left[\prod_{j=1}^{p}\Gamma \left(\frac{t_{j}}{a_{j}}\right)\right]\int_{0}^{\infty }t^{b-\sum_{j=1}^{p}\frac{t_{j}}{a_{j}}-1}e^{-t}dt. \end{array}$$

The result follows. □

### 4.2. Proof for Section 2.1

The corresponding KLD can be expressed as

(2) $$\begin{array}{c} KLD=\log\left[\frac{B\left(b_{1},\ldots ,b_{K-1};b_{K}\right)}{B\left(a_{1},\ldots ,a_{K-1};a_{K}\right)}\right]+\sum_{i=1}^{K}\left(a_{i}-b_{i}\right)E\left[\log X_{i}\right]. \end{array}$$

It is easy to show that

$$\begin{array}{c} E\left[\log X_{i}\right]=\psi \left(a_{i}\right)-\psi \left(a_{1}+\cdots +a_{K}\right), \end{array}$$

so (2) reduces to the required.

### 4.3. Proof for Section 2.2

The corresponding KLD can be expressed as

(3) $$\begin{array}{c} KLD=\log\left[\frac{\alpha \Gamma \left(\frac{p}{\beta }\right)}{\beta \Gamma \left(\frac{p}{\alpha }\right)}\right]+\frac{1}{2}\log\frac{\left|V_{2}\right|}{\left|V_{1}\right|}+E\left[{\left(X^{T}V_{2}^{-1}X\right)}^{\frac{\beta }{2}}\right]-E\left[{\left(X^{T}V_{1}^{-1}X\right)}^{\frac{\alpha }{2}}\right]. \end{array}$$

The second expectation in (3) can be expressed as

$$\begin{array}{cc} E\left[{\left(X^{T}V_{1}^{-1}X\right)}^{\frac{\alpha }{2}}\right] & =\frac{\alpha \Gamma \left(\frac{p}{2}\right)}{2\pi^{\frac{p}{2}}\Gamma \left(\frac{p}{\alpha }\right)}{\left|V_{1}\right|}^{-\frac{1}{2}}\int_{R^{p}}{\left(x^{T}V_{1}^{-1}x\right)}^{\frac{\alpha }{2}}\exp\left\{-{\left(x^{T}V_{1}^{-1}x\right)}^{\frac{\alpha }{2}}\right\}dx\\ \; & =\frac{\alpha \Gamma \left(\frac{p}{2}\right)}{2\pi^{\frac{p}{2}}\Gamma \left(\frac{p}{\alpha }\right)}\int_{R^{p}}{\left(y^{T}y\right)}^{\frac{\alpha }{2}}\exp\left\{-{\left(y^{T}y\right)}^{\frac{\alpha }{2}}\right\}dy\\ \; & =\frac{\alpha }{2\Gamma \left(\frac{p}{\alpha }\right)}\int_{0}^{\infty }u^{\frac{p}{2}+\frac{\alpha }{2}-1}\exp\left(-u^{\frac{\alpha }{2}}\right)du\\ \; & =\frac{1}{\Gamma \left(\frac{p}{\alpha }\right)}\int_{0}^{\infty }t^{\frac{p}{\alpha }}\exp\left(-t\right)dt\\ \; & =\frac{p}{\alpha }, \end{array}$$

where $y=V_{1}^{-\frac{1}{2}}x$, $u=y^{T}y$ and $t=u^{\frac{\alpha }{2}}$.

Let $V=V_{1}^{\frac{1}{2}}V_{2}^{-1}V_{1}^{\frac{1}{2}}$ and $V=PDP^{-1}$, where P is an orthonormal matrix composed of eigenvectors of V and D is a diagonal matrix composed of eigenvalues say $\lambda_{i}$ of V. Then, the first expectation in (3) can be expressed as

(4) $$\begin{array}{cc} E\left[{\left(X^{T}V_{2}^{-1}X\right)}^{\frac{\beta }{2}}\right] & =\frac{\alpha \Gamma \left(\frac{p}{2}\right)}{2\pi^{\frac{p}{2}}\Gamma \left(\frac{p}{\alpha }\right)}\int_{R^{p}}{\left[\mathrm{tr}\;\left({\mathrm{DP}}^{T}{\mathrm{yy}}^{T}P\right)\right]}^{\frac{\beta }{2}}\exp\left\{-{\left(y^{T}y\right)}^{\frac{\alpha }{2}}\right\}dy\\ \; & =\frac{\alpha \Gamma \left(\frac{p}{2}\right)}{2\pi^{\frac{p}{2}}\Gamma \left(\frac{p}{\alpha }\right)}\int_{R^{p}}{\left(x^{T}Dx\right)}^{\frac{\beta }{2}}\exp\left\{-{\left(x^{T}x\right)}^{\frac{\alpha }{2}}\right\}dx\\ \; & =\frac{\alpha \Gamma \left(\frac{p}{2}\right)}{2\pi^{\frac{p}{2}}\Gamma \left(\frac{p}{\alpha }\right)}\int_{R}\cdots \int_{R}{\left(\sum_{i}^{p}\lambda_{i}x_{i}^{2}\right)}^{\frac{\beta }{2}}\exp\left\{-{\left(\sum_{i}^{p}x_{i}^{2}\right)}^{\frac{\alpha }{2}}\right\}dx, \end{array}$$

where $y=V_{1}^{-\frac{1}{2}}x$ and $z=P^{T}y$. Using the pseudo-polar transformation $z_{1}=r\sin\theta_{1}$, $z_{2}=r\cos\theta_{1}$$\sin\theta_{2}$, *…*, $z_{p}=r\cos\theta_{1}$$\cos\theta_{2}$⋯$\cos\theta_{p-1}$ https://en.wikipedia.org/wiki/Polar_coordinate_system (accessed on 1 July 2024), (4) can be expressed as

(5) $$\begin{array}{cc} E\left[{\left(X^{T}V_{2}^{-1}X\right)}^{\frac{\beta }{2}}\right] & =\frac{\alpha \Gamma \left(\frac{p}{2}\right)}{2\pi^{\frac{p}{2}}\Gamma \left(\frac{p}{\alpha }\right)}\int_{0}^{\infty }r^{p-1}\int_{-\frac{\pi }{2}}^{\frac{\pi }{2}}\cdots \int_{-\pi }^{\pi }{\left[r^{2}\left(\lambda_{1}\sin^{2}\theta_{1}+\cdots +\lambda_{p}\cos^{2}\theta_{1}\cdots \cos^{2}\theta_{p-1}\right)\right]}^{\frac{\beta }{2}}\\ \; & \;\;\times \exp\left[-{\left(r^{2}\right)}^{\frac{\alpha }{2}}\right]\left[\prod_{j=1}^{p-1}{\left|\cos\theta_{j}\right|}^{p-j-1}\right]dr\prod_{j=1}^{p-1}d\theta_{j}\\ \; & =\frac{\alpha \Gamma \left(\frac{p}{2}\right)}{\pi^{\frac{p}{2}}\Gamma \left(\frac{p}{\alpha }\right)}\int_{0}^{\infty }r^{p+\beta -1}\exp\left(-r^{\alpha }\right)\int_{0}^{1}\cdots \int_{0}^{1}\prod_{j=1}^{p-1}\left[x_{j}^{\frac{p-j}{2}-1}{\left(1-x_{j}\right)}^{-\frac{1}{2}}\right]\\ \; & \;\;\times {\left[B_{p}\left(x_{1},\ldots ,x_{p-1}\right)\right]}^{\frac{\beta }{2}}dx_{1}\cdots dx_{p-1}dr\\ \; & =\frac{\Gamma \left(\frac{p}{2}\right)\Gamma \left(\frac{p+\beta }{2}\right)}{\pi^{\frac{p}{2}}\Gamma \left(\frac{p}{\alpha }\right)}\int_{0}^{1}\cdots \int_{0}^{1}\left\{\prod_{j=1}^{p-1}\left[x_{j}^{\frac{p-j}{2}-1}{\left(1-x_{j}\right)}^{-\frac{1}{2}}\right]\right\}{\left[B_{p}\left(x_{1},\ldots ,x_{p-1}\right)\right]}^{\frac{\beta }{2}}dx_{1}\cdots dx_{p-1}, \end{array}$$

where $x_{i}=\cos^{2}\theta_{i}$ and $B_{p}\left(x_{1},\ldots ,x_{p-1}\right)=\lambda_{1}+\left(\lambda_{2}-\lambda_{1}\right)x_{1}+\cdots +\left(\lambda_{p}-\lambda_{p-1}\right)x_{1}x_{2}\cdots x_{p-1}$. Provided that $\left|\lambda_{1}\right|\geq \left|\left(\lambda_{2}-\lambda_{1}\right)x_{1}+\cdots +\left(\lambda_{p}-\lambda_{p-1}\right)x_{1}x_{2}\cdots x_{p-1}\right|$ holds, we can apply the generalized multinomial theorem to calculate (5) as

EXTV2−1Xβ2=Γp2Γp+β2πp2Γpα∫01⋯∫01∏j=1p−1xjp−j2−11−xj−12×∑r1=0∞∑r2=0r1⋯∑rp−1=0rp−2β2r1r1r2⋯rp−2rp−1×λ1β2−r1λ2−λ1x1r1−r2λ3−λ2x1x2r2−r3⋯λp−1−λp−2x1x2⋯xp−2rp−2−rp−1λp−λp−1x1x2⋯xp−1rp−1dx1⋯dxp−1=Γp2Γp+β2πp2Γpα∫01⋯∫01∏j=1p−1xjp−j2−11−xj−12×∑r1=0∞∑r2=0r1⋯∑rp−1=0rp−2β2r1r1r2⋯rp−2rp−1λ1β2−r1×∏j=1p−1λj+1−λj∏ℓ=1jxℓrj−rj+1dx1⋯dxp−1=Γp2Γp+β2πp2Γpα∑r1=0∞∑r2=0r1⋯∑rp−1=0rp−2β2r1r1r2⋯rp−2rp−1λ1β2−r1×∏j=1p−1λj+1−λjrj−rj+1Brj+p−j2,12

provided that the infinite sum converges. Hence, the required.

### 4.4. Proof for Section 2.3

The corresponding KLD can be expressed as

(6) $$\begin{array}{cc} \; & KLD=\log\left[\frac{\Gamma \left(a_{1}+\cdots +a_{K+1}\right)\Gamma \left(b_{1}\right)\cdots \Gamma \left(b_{K+1}\right)}{\Gamma \left(b_{1}+\cdots +b_{K+1}\right)\Gamma \left(a_{1}\right)\cdots \Gamma \left(a_{K+1}\right)}\right]+\sum_{i=1}^{K}\left(a_{i}-b_{i}\right)E\left[\log X_{i}\right]\\ \; & \;\;\;+\left(b_{1}+\cdots +b_{K+1}-a_{1}-\cdots -a_{K+1}\right)E\left[\log\left(1+\sum_{i=1}^{K}X_{i}\right)\right]. \end{array}$$

It is easy to show that

$$\begin{array}{c} E\left[\log X_{i}\right]=\psi \left(a_{i}\right)-\psi \left(a_{K+1}\right) \end{array}$$

and

$$\begin{array}{c} E\left[\log\left(1+\sum_{i=1}^{K}X_{i}\right)\right]=\psi \left(a_{1}+\cdots +a_{K+1}\right)-\psi \left(a_{K+1}\right), \end{array}$$

so (6) reduces to the required.

### 4.5. Proof for Section 2.4

The corresponding KLD can be expressed as

(7) $$\begin{array}{cc} \; & KLD=\log\left[\frac{C\left(a_{1},\ldots ,a_{K},b,c\right)}{C\left(d_{1},\ldots ,d_{K},e,f\right)}\right]+\sum_{i=1}^{K}\left(a_{i}-d_{i}\right)E\left[\log X_{i}\right]+\left(b-e\right)E\left[\log\left(1-\sum_{i=1}^{K}X_{i}\right)\right]\\ \; & \;\;\;-\left(c-f\right)E\left[\log\left(1+\sum_{i=1}^{K}X_{i}\right)\right]. \end{array}$$

It is easy to show that

$$\begin{array}{c} E\left[\log X_{i}\right]={\left.\frac{\partial }{\partial \alpha }\left[\frac{C\left(a_{1},\ldots ,a_{i},\ldots ,a_{K},b,c\right)}{C\left(a_{1},\ldots ,a_{i}+\alpha ,\ldots ,a_{K},b,c\right)}\right]\right|}_{\alpha =0}, \end{array}$$

$$\begin{array}{c} E\left[\log\left(1-\sum_{i=1}^{K}X_{i}\right)\right]={\left.\frac{\partial }{\partial \alpha }\left[\frac{C\left(a_{1},\ldots ,a_{i},\ldots ,a_{K},b,c\right)}{C\left(a_{1},\ldots ,a_{K},b+\alpha ,c\right)}\right]\right|}_{\alpha =0} \end{array}$$

and

$$\begin{array}{c} E\left[\log\left(1+\sum_{i=1}^{K}X_{i}\right)\right]={\left.\frac{\partial }{\partial \alpha }\left[\frac{C\left(a_{1},\ldots ,a_{i},\ldots ,a_{K},b,c\right)}{C\left(a_{1},\ldots ,a_{K},b,c-\alpha \right)}\right]\right|}_{\alpha =0}, \end{array}$$

so (7) reduces to the required.

### 4.6. Proof for Section 2.5

The corresponding KLD can be expressed as

(8) $$\begin{array}{cc} \; & KLD=\log\left[\frac{aq^{\frac{2N+p-2}{2a}}\Gamma \left(\frac{2M+p-2}{2b}\right)}{bs^{\frac{2M+p-2}{2b}}\Gamma \left(\frac{2N+p-2}{2a}\right)}\right]+\frac{1}{2}\log\frac{\left|\Sigma_{2}\right|}{\left|\Sigma_{1}\right|}+\left(N-1\right)E\left[\log\left(X^{T}\Sigma_{1}^{-1}X\right)\right]\\ \; & \;\;\;-qE\left[{\left(X^{T}\Sigma_{1}^{-1}X\right)}^{a}\right]-\left(M-1\right)E\left[\log\left(X^{T}\Sigma_{2}^{-1}X\right)\right]+sE\left[{\left(X^{T}\Sigma_{2}^{-1}X\right)}^{b}\right]. \end{array}$$

The second expectation in (8) can be calculated as

$$\begin{array}{cc} E\left[{\left(X^{T}\Sigma_{1}^{-1}X\right)}^{a}\right] & =\frac{a\Gamma \left(\frac{p}{2}\right)q^{\frac{2N+p-2}{2a}}}{\pi^{\frac{p}{2}}\Gamma \left(\frac{2N+p-2}{2a}\right)}{\left|\Sigma_{1}\right|}^{-\frac{1}{2}}\int_{R^{p}}{\left(x^{T}\Sigma_{1}^{-1}x\right)}^{a+N-1}\exp\left\{-q{\left(x^{T}\Sigma_{1}^{-1}x\right)}^{a}\right\}dx\\ \; & =\frac{a\Gamma \left(\frac{p}{2}\right)q^{\frac{2N+p-2}{2a}}}{\pi^{\frac{p}{2}}\Gamma \left(\frac{2N+p-2}{2a}\right)}\int_{R^{p}}{\left(y^{T}y\right)}^{a+N-1}\exp\left\{-q{\left(y^{T}y\right)}^{a}\right\}dy\\ \; & =\frac{1}{q\Gamma \left(\frac{2N+p-2}{2a}\right)}\int_{0}^{\infty }t^{\frac{2N+p-2}{2a}}\exp\left(-t\right)dt\\ \; & =\frac{2N+p-2}{2aq}, \end{array}$$

where $y=\Sigma_{1}^{-\frac{1}{2}}x$ and $t=q{\left(y^{T}y\right)}^{a}$.

Let $\Sigma =\Sigma_{1}^{\frac{1}{2}}\Sigma_{2}^{-1}\Sigma_{1}^{\frac{1}{2}}$. The first expectation in (8) can be calculated as

$$\begin{array}{cc} E\left[\log\left(X^{T}\Sigma_{1}^{-1}X\right)\right] & =\frac{a\Gamma \left(\frac{p}{2}\right)q^{\frac{2N+p-2}{2a}}}{\pi^{\frac{p}{2}}\Gamma \left(\frac{2N+p-2}{2a}\right)}{\left|\Sigma_{1}\right|}^{-\frac{1}{2}}\int_{R^{p}}{\left(x^{T}\Sigma_{1}^{-1}x\right)}^{N-1}\log\left(x^{T}\Sigma_{1}^{-1}x\right)\exp\left\{-q{\left(x^{T}\Sigma_{1}^{-1}x\right)}^{a}\right\}dx\\ \; & =\frac{a\Gamma \left(\frac{p}{2}\right)q^{\frac{2N+p-2}{2a}}}{\pi^{\frac{p}{2}}\Gamma \left(\frac{2N+p-2}{2a}\right)}\int_{R^{p}}{\left(y^{T}y\right)}^{N-1}\log\left(y^{T}y\right)\exp\left\{-q{\left(y^{T}y\right)}^{a}\right\}dy\\ \; & =\frac{a\Gamma \left(\frac{p}{2}\right)q^{\frac{2N+p-2}{2a}}}{\pi^{\frac{p}{2}}\Gamma \left(\frac{2N+p-2}{2a}\right)}\frac{\partial }{\partial N}\int_{R^{p}}{\left(y^{T}y\right)}^{N-1}\exp\left\{-q{\left(y^{T}y\right)}^{a}\right\}dy\\ \; & =\frac{q^{\frac{2N+p-2}{2a}}}{\Gamma \left(\frac{2N+p-2}{2a}\right)}\frac{\partial }{\partial N}\int_{0}^{\infty }\frac{t^{\frac{2N+p-2}{2a}-1}}{q^{\frac{2N+p-2}{2a}}}\exp\left(-t\right)dt\\ \; & =\frac{q^{\frac{2N+p-2}{2a}}}{\Gamma \left(\frac{2N+p-2}{2a}\right)}\frac{\partial }{\partial N}\left[\frac{\Gamma \left(\frac{2N+p-2}{2a}\right)}{q^{\frac{2N+p-2}{2a}}}\right]\\ \; & =\frac{1}{a}\left[\psi \left(\frac{2N+p-2}{2a}\right)-\log q\right], \end{array}$$

where $y=\Sigma_{1}^{-\frac{1}{2}}x$ and $t=q{\left(y^{T}y\right)}^{a}$.

As in Section 4.3, write $\Sigma =PDP^{-1}$, where P is an orthonormal matrix composed of eigenvectors of Σ and D is a diagonal matrix composed of eigenvalues say $\lambda_{i}$ of Σ. Then, the fourth expectation in (8) can be expressed as

(9) $$\begin{array}{cc} E\left[{\left(X^{T}\Sigma_{2}^{-1}X\right)}^{b}\right] & =\frac{a\Gamma \left(\frac{p}{2}\right)q^{\frac{2N+p-2}{2a}}}{\pi^{\frac{p}{2}}\Gamma \left(\frac{2N+p-2}{2a}\right)}{\left|\Sigma_{1}\right|}^{-\frac{1}{2}}\int_{R^{p}}{\left(x^{T}\Sigma_{2}^{-1}x\right)}^{b}{\left(x^{T}\Sigma_{1}^{-1}x\right)}^{N-1}\exp\left\{-q{\left(x^{T}\Sigma_{1}^{-1}x\right)}^{a}\right\}dx\\ \; & =\frac{a\Gamma \left(\frac{p}{2}\right)q^{\frac{2N+p-2}{2a}}}{\pi^{\frac{p}{2}}\Gamma \left(\frac{2N+p-2}{2a}\right)}\int_{R^{p}}{\left(y^{T}\Sigma y\right)}^{b}{\left(y^{T}y\right)}^{N-1}\exp\left\{-q{\left(y^{T}y\right)}^{a}\right\}dy\\ \; & =\frac{a\Gamma \left(\frac{p}{2}\right)q^{\frac{2N+p-2}{2a}}}{\pi^{\frac{p}{2}}\Gamma \left(\frac{2N+p-2}{2a}\right)}\int_{R^{p}}{\left[\mathrm{tr}\;\left(DP^{T}yy^{T}P\right)\right]}^{b}{\left(y^{T}y\right)}^{N-1}\exp\left\{-q{\left(y^{T}y\right)}^{a}\right\}dy\\ \; & =\frac{a\Gamma \left(\frac{p}{2}\right)q^{\frac{2N+p-2}{2a}}}{\pi^{\frac{p}{2}}\Gamma \left(\frac{2N+p-2}{2a}\right)}\int_{R^{p}}{\left(v^{T}Dv\right)}^{b}{\left(v^{T}v\right)}^{N-1}\exp\left\{-q{\left(v^{T}v\right)}^{a}\right\}dv\\ \; & =\frac{a\Gamma \left(\frac{p}{2}\right)q^{\frac{2N+p-2}{2a}}}{\pi^{\frac{p}{2}}\Gamma \left(\frac{2N+p-2}{2a}\right)}\int_{R^{p}}{\left(\sum_{i=1}^{p}\lambda_{i}v_{i}^{2}\right)}^{b}{\left(\sum_{i=1}^{p}v_{i}^{2}\right)}^{N-1}\exp\left\{-q{\left(\sum_{i=1}^{p}v_{i}^{2}\right)}^{a}\right\}dv, \end{array}$$

where $y=\Sigma_{1}^{-\frac{1}{2}}x$ and $v=P^{T}y$.

Using the pseudo-polar transformation $v_{1}=r\sin\theta_{1}$, $v_{2}=r\cos\theta_{1}\sin\theta_{2}$, *…*, $v_{p}=r$$\cos\theta_{1}$$\cos\theta_{2}$⋯$\cos\theta_{p-1}$, (9) can be expressed as

(10) $$\begin{array}{cc} E\left[{\left(X^{T}\Sigma_{2}^{-1}X\right)}^{b}\right] & =\frac{a\Gamma \left(\frac{p}{2}\right)q^{\frac{2N+p-2}{2a}}}{\pi^{\frac{p}{2}}\Gamma \left(\frac{2N+p-2}{2a}\right)}\int_{0}^{\infty }r^{p-1}\int_{-\frac{\pi }{2}}^{\frac{\pi }{2}}\cdots \int_{-\pi }^{\pi }{\left[r^{2}\left(\lambda_{1}\sin^{2}\theta_{1}+\cdots +\lambda_{p}\cos^{2}\theta_{1}\cdots \cos^{2}\theta_{p-1}\right)\right]}^{b}\\ \; & \;\;\;\times {\left(r^{2}\right)}^{N-1}\exp\left[-q{\left(r^{2}\right)}^{a}\right]\left[\prod_{j=1}^{p-1}{\left|\cos\theta_{j}\right|}^{p-j-1}\right]dr\prod_{j=1}^{p-1}d\theta_{j}\\ \; & =\frac{\Gamma \left(\frac{p}{2}\right)q^{\frac{2N+p-2}{2a}}}{\pi^{\frac{p}{2}}\Gamma \left(\frac{2N+p-2}{2a}\right)}\int_{0}^{\infty }\frac{t^{\frac{2N+p+2b-2}{2a}-1}}{q^{\frac{2N+p+2b-2}{2a}}}\exp\left(-t\right)\int_{0}^{1}\cdots \int_{0}^{1}\left\{\prod_{j=1}^{p-1}\left[x_{j}^{\frac{p-j}{2}-1}{\left(1-x_{j}\right)}^{-\frac{1}{2}}\right]\right\}\\ \; & \;\;\;\times {\left[B_{p}\left(x_{1},\ldots ,x_{p-1}\right)\right]}^{b}dx_{1}\cdots dx_{p-1}dt\\ \; & =\frac{\Gamma \left(\frac{p}{2}\right)\Gamma \left(\frac{2N+p+2b-2}{2a}\right)}{\pi^{\frac{p}{2}}q^{\frac{b}{a}}\Gamma \left(\frac{2N+p-2}{2a}\right)}\int_{0}^{1}\cdots \int_{0}^{1}\left\{\prod_{j=1}^{p-1}\left[x_{j}^{\frac{p-j}{2}-1}{\left(1-x_{j}\right)}^{-\frac{1}{2}}\right]\right\}\\ \; & \;\;\;\times {\left[B_{p}\left(x_{1},\ldots ,x_{p-1}\right)\right]}^{b}dx_{1}\cdots dx_{p-1}, \end{array}$$

where $x_{i}=\cos^{2}\theta_{i}$, $t=qr^{2a}$ and $B_{p}\left(x_{1},\ldots ,x_{p-1}\right)=\lambda_{1}+\left(\lambda_{2}-\lambda_{1}\right)x_{1}+\cdots +\left(\lambda_{p}-\lambda_{p-1}\right)$$x_{1}x_{2}\cdots x_{p-1}$.

Provided that $\left|\lambda_{1}\right|\geq \left|\left(\lambda_{2}-\lambda_{1}\right)x_{1}+\cdots +\left(\lambda_{p}-\lambda_{p-1}\right)x_{1}x_{2}\cdots x_{p-1}\right|$ holds, we can apply the generalized multinomial theorem to calculate (10) as

EXTΣ2−1Xb=Γp2Γ2N+p+2b−22aπp2qbaΓ2N+p−22a∫01⋯∫01∏j=1p−1xjp−j2−11−xj−12×∑r1=0∞∑r2=0r1⋯∑rp−1=0rp−2br1r1r2⋯rp−2rp−1λ1b−r1λ2−λ1x1r1−r2λ3−λ2x1x2r2−r3⋯λp−1−λp−2x1x2⋯xp−2rp−2−rp−1λp−λp−1x1x2⋯xp−1rp−1dx1⋯dxp−1=Γp2Γ2N+p+2b−22aπp2qbaΓ2N+p−22a∫01⋯∫01∏j=1p−1xjp−j2−11−xj−12×∑r1=0∞∑r2=0r1⋯∑rp−1=0rp−2br1r1r2⋯rp−2rp−1×λ1b−r1∏j=1p−1λj+1−λj∏ℓ=1jxℓrj−rj+1dx1⋯dxp−1=Γp2Γ2N+p+2b−22aπp2qbaΓ2N+p−22a∑r1=0∞∑r2=0r1⋯∑rp−1=0rp−2br1r1r2⋯rp−2rp−1λ1b−r1×∏j=1p−1λj+1−λjrj−rj+1Brj+p−j2,12

provided that the infinite sum converges.

The third expectation in (8) can be expressed as

$$\begin{array}{cc} E\left[\log\left(X^{T}\Sigma_{2}^{-1}X\right)\right] & =\frac{a\Gamma \left(\frac{p}{2}\right)q^{\frac{2N+p-2}{2a}}}{\pi^{\frac{p}{2}}\Gamma \left(\frac{2N+p-2}{2a}\right)}{\left|\Sigma_{1}\right|}^{-\frac{1}{2}}\int_{R^{p}}{\left(x^{T}\Sigma_{1}^{-1}x\right)}^{N-1}\log\left(x^{T}\Sigma_{2}^{-1}x\right)\exp\left\{-q{\left(x^{T}\Sigma_{1}^{-1}x\right)}^{a}\right\}dx\\ \; & =\frac{a\Gamma \left(\frac{p}{2}\right)q^{\frac{2N+p-2}{2a}}}{\pi^{\frac{p}{2}}\Gamma \left(\frac{2N+p-2}{2a}\right)}\int_{R^{p}}{\left(y^{T}y\right)}^{N-1}\log\left[\mathrm{tr}\;\left(DP^{T}yy^{T}P\right)\right]\exp\left\{-q{\left(y^{T}y\right)}^{a}\right\}dy\\ \; & =\frac{a\Gamma \left(\frac{p}{2}\right)q^{\frac{2N+p-2}{2a}}}{\pi^{\frac{p}{2}}\Gamma \left(\frac{2N+p-2}{2a}\right)}\int_{R^{p}}{\left(\sum_{i=1}^{p}v_{i}^{2}\right)}^{N-1}\log\left(\sum_{i=1}^{p}\lambda_{i}v_{i}^{2}\right)\exp\left\{-q{\left(\sum_{i=1}^{p}v_{i}^{2}\right)}^{a}\right\}dv\\ \; & =\frac{a\Gamma \left(\frac{p}{2}\right)q^{\frac{2N+p-2}{2a}}}{\pi^{\frac{p}{2}}\Gamma \left(\frac{2N+p-2}{2a}\right)}\int_{0}^{\infty }r^{p-1}\int_{-\frac{\pi }{2}}^{\frac{\pi }{2}}\cdots \int_{-\pi }^{\pi }{\left(r^{2}\right)}^{N-1}\\ \; & \;\times \log\left[r^{2}\left(\lambda_{1}\sin^{2}\theta_{1}+\cdots +\lambda_{p}\cos^{2}\theta_{1}\cdots \cos^{2}\theta_{p-1}\right)\right]\\ \; & \;\times \exp\left\{-q{\left(r^{2}\right)}^{a}\right\}\left[\prod_{j=1}^{p-1}{\left|\cos\theta_{j}\right|}^{p-j-1}\right]dr\prod_{j=1}^{p-1}d\theta_{j}\\ \; & =I_{1}+I_{2} \end{array}$$

say, where $y=\Sigma_{1}^{-\frac{1}{2}}x$, $v=P^{T}y$,

$$\begin{array}{cc} I_{1}=\frac{2a\Gamma \left(\frac{p}{2}\right)q^{\frac{2N+p-2}{2a}}}{\pi^{\frac{p}{2}}\Gamma \left(\frac{2N+p-2}{2a}\right)}\int_{0}^{\infty }r^{p+2N-3}\log\left(r^{2}\right)\exp\left(-qr^{2a}\right)\\ \; & \times \int_{0}^{1}\cdots \int_{0}^{1}\prod_{j=1}^{p-1}\left[x_{j}^{\frac{p-j}{2}-1}{\left(1-x_{j}\right)}^{-\frac{1}{2}}\right]dx_{1}\cdots dx_{p-1}dr \end{array}$$

and

$$\begin{array}{cc} I_{2}=\frac{2a\Gamma \left(\frac{p}{2}\right)q^{\frac{2N+p-2}{2a}}}{\pi^{\frac{p}{2}}\Gamma \left(\frac{2N+p-2}{2a}\right)}\int_{0}^{\infty }r^{p+2N-3}\exp\left(-qr^{2a}\right)\\ \; & \;\times \int_{0}^{1}\cdots \int_{0}^{1}\left\{\prod_{j=1}^{p-1}\left[x_{j}^{\frac{p-j}{2}-1}{\left(1-x_{j}\right)}^{-\frac{1}{2}}\right]\right\}\log\left[B_{p}\left(x_{1},\ldots ,x_{p-1}\right)\right]dx_{1}\cdots dx_{p-1}dr. \end{array}$$

The $I_{1}$ can be calculated as

$$\begin{array}{cc} I_{1}\; & =\frac{\Gamma \left(\frac{p}{2}\right)q^{\frac{2N+p-2}{2a}}}{\pi^{\frac{p}{2}}\Gamma \left(\frac{2N+p-2}{2a}\right)}\int_{0}^{\infty }\frac{t^{\frac{p+2N-2}{2a}-1}}{q^{\frac{2N+p-2}{2a}}}\log{\left(\frac{t}{q}\right)}^{\frac{1}{a}}\exp\left(-t\right)\prod_{j=1}^{p-1}B\left(\frac{p-j}{2},\frac{1}{2}\right)dt\\ \; & =\frac{\Gamma \left(\frac{p}{2}\right)}{a\pi^{\frac{p}{2}}\Gamma \left(\frac{2N+p-2}{2a}\right)}\left\{a\frac{\partial }{\partial N}\int_{0}^{\infty }t^{\frac{p+2N-2}{2a}-1}\exp\left(-t\right)-\Gamma \left(\frac{2N+p-2}{2a}\right)\log q\right\}\prod_{j=1}^{p-1}B\left(\frac{p-j}{2},\frac{1}{2}\right)\\ \; & =\frac{\Gamma \left(\frac{p}{2}\right)}{a\pi^{\frac{p}{2}}\Gamma \left(\frac{2N+p-2}{2a}\right)}\left\{a\frac{\partial }{\partial N}\Gamma \left(\frac{p+2N-2}{2a}\right)-\Gamma \left(\frac{2N+p-2}{2a}\right)\log q\right\}\prod_{j=1}^{p-1}B\left(\frac{p-j}{2},\frac{1}{2}\right)\\ \; & =\frac{\Gamma \left(\frac{p}{2}\right)}{a\pi^{\frac{p}{2}}}\left\{\psi \left(\frac{p+2N-2}{2a}\right)-\log q\right\}\prod_{j=1}^{p-1}B\left(\frac{p-j}{2},\frac{1}{2}\right), \end{array}$$

where $t=qr^{2a}$.

The $I_{2}$ can be calculated as

I2=Γp2q2N+p−22aπp2Γ2N+p−22a∫0∞t2N+p−22a−1q2N+p−22aexp(−t)×∫01⋯∫01∏j=1p−1xjp−j2−11−xj−12logBpx1,…,xp−1dx1⋯dxp−1dt=Γp2πp2∫01⋯∫01∏j=1p−1xjp−j2−11−xj−12×logλ1+log1+λ2−λ1x1λ1+⋯+λp−λp−1x1x2⋯xp−1λ1dx1⋯dxp−1=Γp2πp2logλ1∏j=1p−1Bp−j2,12−Γp2πp2∫01⋯∫01∏j=1p−1xjp−j2−11−xj−12×∑k=1∞(−1)kkλ2−λ1x1λ1+⋯+λp−λp−1x1x2⋯xp−1λ1kdx1⋯dxp−1=Γp2πp2logλ1∏j=1p−1Bp−j2,12−Γp2πp2∑k=1∞(−1)kk∑i1+⋯+ip−1=kki1,…,ip−1×∫01⋯∫01∏j=1p−1xjp−j2−11−xj−12λj+1−λjλ1∏ℓ=1jxℓijdx1⋯dxp−1=Γp2πp2logλ1∏j=1p−1Bp−j2,12−Γp2πp2∑k=1∞(−1)kk∑i1+⋯+ip−1=kki1,…,ip−1×∏j=1p−1λj+1−λjλ1ijB∑ℓ=jp−1iℓ+p−j2,12

provided that the infinite sum converges.

Hence, the required.

### 4.7. Proof for Section 2.6

The corresponding KLD can be expressed as

(11) $$\begin{array}{cc} KLD & =\log\left(\frac{a_{1}\cdots a_{p}}{b_{1}\cdots b_{p}}\right)+\sum_{\ell =1}^{p}\left(b_{\ell }-a_{\ell }\right)E\left(X_{\ell }\right)\\ \; & \;+\left(p+1\right)E\left\{\log\left[1+\exp\left(-b_{1}X_{1}\right)+\cdots +\exp\left(-b_{p}X_{p}\right)\right]\right\}\\ \; & \;-\left(p+1\right)E\left\{\log\left[1+\exp\left(-a_{1}X_{1}\right)+\cdots +\exp\left(-a_{p}X_{p}\right)\right]\right\}\\ \; & =\log\left(\frac{a_{1}\cdots a_{p}}{b_{1}\cdots b_{p}}\right)+\left(p+1\right)E\left\{\log\left[1+\exp\left(-b_{1}X_{1}\right)+\cdots +\exp\left(-b_{p}X_{p}\right)\right]\right\}\\ \; & \;-\left(p+1\right)E\left\{\log\left[1+\exp\left(-a_{1}X_{1}\right)+\cdots +\exp\left(-a_{p}X_{p}\right)\right]\right\} \end{array}$$

since the expectations are zero. Using the Taylor expansion for log(1+z), the first expectation in (11) can be expressed as

−∑k=1∞(−1)kkEexp−b1X1+⋯+exp−bpXpk=−∑k=1∞(−1)kk∑i1+⋯+ip=kki1,…,ipEexp−i1b1X1−⋯−ipbpXp=−∑k=1∞(−1)kk∑i1+⋯+ip=kki1,…,ip∏j=1pΓaj+ijbjajΓ1−∑j=1pijbjaj,

where the last step follows by Lemma 1 provided that $\sum_{j=1}^{p}\frac{i_{j}b_{j}}{a_{j}}<1$ and the infinite series converges. The second expectation in (11) can be expressed as

$$\begin{array}{cc} \; & p!a_{1}\cdots a_{p}\int_{R^{p}}\left\{{\left.\frac{\partial }{\partial \alpha }{\left[1+\exp\left(-a_{1}x_{1}\right)+\cdots +\exp\left(-a_{p}x_{p}\right)\right]}^{\alpha }\right|}_{\alpha =0}\right\}\\ \; & \;\;\;\times \frac{\exp\left(-a_{1}x_{1}-\cdots -a_{p}x_{p}\right)dx_{1}\cdots dx_{p}}{{\left[1+\exp\left(-a_{1}x_{1}\right)+\cdots +\exp\left(-a_{p}x_{p}\right)\right]}^{p+1}}\\ \; & =p!a_{1}\cdots a_{p}\frac{\partial }{\partial \alpha }{\left.\left\{\int_{R^{p}}\frac{\exp\left(-a_{1}x_{1}-\cdots -a_{p}x_{p}\right)}{{\left[1+\exp\left(-a_{1}x_{1}\right)+\cdots +\exp\left(-a_{p}x_{p}\right)\right]}^{p+1-\alpha }}dx_{1}\cdots dx_{p}\right\}\right|}_{\alpha =0}\\ \; & =p!\frac{\partial }{\partial \alpha }{\left.\left[\frac{\Gamma (1-\alpha )}{\Gamma (p+1-\alpha )}\right]\right|}_{\alpha =0}\\ \; & =\frac{\Gamma^{\prime }\left(p+1\right)-\Gamma \left(p+1\right)\Gamma^{\prime }\left(1\right)}{p!}, \end{array}$$

where the penultimate step is followed by Lemma 1. Hence, the required.

### 4.8. Proof for Section 2.7

The corresponding KLD can be expressed as

(12) $$\begin{array}{cc} KLD & =\log\left[\frac{{\left(b\right)}_{p}a_{1}\cdots a_{p}}{{\left(d\right)}_{p}b_{1}\cdots b_{p}}\right]+\sum_{\ell =1}^{p}\left(c_{\ell }-a_{\ell }\right)E\left(X_{\ell }\right)\\ \; & \;+\left(d+p\right)E\left\{\log\left[1+\exp\left(-c_{1}X_{1}\right)+\cdots +\exp\left(-c_{p}X_{p}\right)\right]\right\}\\ \; & \;-\left(b+p\right)E\left\{\log\left[1+\exp\left(-a_{1}X_{1}\right)+\cdots +\exp\left(-a_{p}X_{p}\right)\right]\right\}\\ \; & =\log\left[\frac{{\left(b\right)}_{p}a_{1}\cdots a_{p}}{{\left(d\right)}_{p}b_{1}\cdots b_{p}}\right]+\left(d+p\right)E\left\{\log\left[1+\exp\left(-c_{1}X_{1}\right)+\cdots +\exp\left(-c_{p}X_{p}\right)\right]\right\}\\ \; & \;-\left(b+p\right)E\left\{\log\left[1+\exp\left(-a_{1}X_{1}\right)+\cdots +\exp\left(-a_{p}X_{p}\right)\right]\right\} \end{array}$$

since the expectations are zero. Using the Taylor expansion for log(1+z), the first expectation in (12) can be expressed as

−∑k=1∞(−1)kkEexp−c1X1+⋯+exp−cpXpk=−∑k=1∞(−1)kk∑i1+⋯+ip=kki1,…,ipEexp−i1c1X1−⋯−ipcpXp=−1Γ(b)∑k=1∞(−1)kk∑i1+⋯+ip=kki1,…,ip∏j=1pΓaj+ijcjajΓb−∑j=1pijcjaj,

where the last step follows by Lemma 1 provided that $\sum_{j=1}^{p}\frac{i_{j}c_{j}}{a_{j}}<b$ and the infinite series converges. The second expectation in (12) can be expressed as

$$\begin{array}{cc} \; & {\left(b\right)}_{p}a_{1}\cdots a_{p}\int_{R^{p}}\left\{{\left.\frac{\partial }{\partial \alpha }{\left[1+\exp\left(-a_{1}x_{1}\right)+\cdots +\exp\left(-a_{p}x_{p}\right)\right]}^{\alpha }\right|}_{\alpha =0}\right\}\\ \; & \;\;\;\times \frac{\exp\left(-a_{1}x_{1}-\cdots -a_{p}x_{p}\right)dx_{1}\cdots dx_{p}}{{\left[1+\exp\left(-a_{1}x_{1}\right)+\cdots +\exp\left(-a_{p}x_{p}\right)\right]}^{b+p}}\\ \; & ={\left(b\right)}_{p}a_{1}\cdots a_{p}\frac{\partial }{\partial \alpha }{\left.\left\{\int_{R^{p}}\frac{\exp\left(-a_{1}x_{1}-\cdots -a_{p}x_{p}\right)}{{\left[1+\exp\left(-a_{1}x_{1}\right)+\cdots +\exp\left(-a_{p}x_{p}\right)\right]}^{b+p-\alpha }}dx_{1}\cdots dx_{p}\right\}\right|}_{\alpha =0}\\ \; & ={\left(b\right)}_{p}\frac{\partial }{\partial \alpha }{\left.\left[\frac{\Gamma (b-\alpha )}{\Gamma (b+p-\alpha )}\right]\right|}_{\alpha =0}\\ \; & =\frac{\Gamma \left(b\right)\Gamma^{\prime }\left(b+p\right)-\Gamma \left(b+p\right)\Gamma^{\prime }\left(b\right)}{\Gamma (b)\Gamma (b+p)}, \end{array}$$

where the penultimate step is followed by Lemma 1. Hence, the required.

### 4.9. Proof for Section 2.8

The corresponding KLD can be expressed as

(13) $$\begin{array}{cc} KLD & =\log\left[\frac{\sqrt{a_{1}\cdots a_{p}}\beta_{p}\left(c,a_{1},\ldots ,a_{p}\right)}{\sqrt{b_{1}\cdots b_{p}}\beta_{p}\left(d,b_{1},\ldots ,b_{p}\right)}\right]\\ \; & \;+\frac{1}{2}\sum_{i=1}^{p}\left(b_{i}-a_{i}\right)E\left(X_{i}^{2}\right)\\ \; & \;+\frac{1}{2}\left(db_{1}\cdots b_{p}-ca_{1}\cdots a_{p}\right)E\left(X_{1}^{2}\cdots X_{p}^{2}\right). \end{array}$$

Using results in [8], we can calculate (13) as the required.

### 4.10. Proof for Section 2.9

The corresponding KLD can be expressed as

(14) $$\begin{array}{cc} \; & KLD=\log\left[\frac{\Gamma \left(\frac{K}{2}+a+b-1\right)\Gamma \left(c\right)\Gamma \left(\frac{K}{2}+d-1\right)}{\Gamma \left(a\right)\Gamma \left(\frac{K}{2}+b-1\right)\Gamma \left(\frac{K}{2}+c+d-1\right)}\right]+\left(b-d\right)E\left[\log\left(\sum_{i=1}^{K}X_{i}^{2}\right)\right]\\ \; & \;\;\;+\left(a-c\right)E\left[\log\left(1-\sum_{i=1}^{K}X_{i}^{2}\right)\right]. \end{array}$$

It is easy to show that

$$\begin{array}{c} E\left[\log\left(\sum_{i=1}^{K}X_{i}^{2}\right)\right]=\psi \left(\frac{K}{2}+b-1\right)-\psi \left(\frac{K}{2}+a+b-1\right) \end{array}$$

and

$$\begin{array}{c} E\left[\log\left(1-\sum_{i=1}^{K}X_{i}^{2}\right)\right]=\psi \left(a\right)-\psi \left(\frac{K}{2}+a+b-1\right), \end{array}$$

so (14) reduces to the required.

### 4.11. Proof for Section 2.10

The corresponding KLD can be expressed as

(15) $$\begin{array}{cc} \; & KLD=\log\left[\frac{C\left(d,e,f\right)}{C\left(a,b,c\right)}\right]+\left(a-d\right)E\left[\log\left(\prod_{i=1}^{p}X_{i}\right)\right]+\left(b-e\right)E\left[\log\left(\prod_{i=1}^{p}\left(1-X_{i}\right)\right)\right]\\ \; & \;\;\;+2\left(c-f\right)E\left[\log\left(\prod_{1\leq i<j\leq p}\left(X_{i}-X_{j}\right)\right)\right]. \end{array}$$

Easy calculations show that

$$\begin{array}{c} E\left[\log\left(\prod_{i=1}^{p}X_{i}\right)\right]={\left.\frac{\partial }{\partial \alpha }\left[\frac{C(a,b,c)}{C(a+\alpha ,b,c)}\right]\right|}_{\alpha =0}, \end{array}$$

$$\begin{array}{c} E\left[\log\left(\prod_{i=1}^{p}\left(1-X_{i}\right)\right)\right]={\left.\frac{\partial }{\partial \alpha }\left[\frac{C(a,b,c)}{C(a,b+\alpha ,c)}\right]\right|}_{\alpha =0} \end{array}$$

and

$$\begin{array}{c} E\left[\log\left(\prod_{1\leq i<j\leq p}\left(X_{i}-X_{j}\right)\right)\right]={\left.\frac{\partial }{\partial \alpha }\left[\frac{C(a,b,c)}{C(a,b,c+\alpha )}\right]\right|}_{\alpha =0}, \end{array}$$

so (7) reduces to the required.

### 4.12. Proof for Section 2.11

The corresponding KLD can be expressed as

(16) $$\begin{array}{cc} \; & KLD=\log\left[\frac{b_{p+1}\left(\sum_{i=1}^{p}a_{i}\right)\left(\prod_{i=1}^{p}a_{i}\right)}{a_{p+1}\left(\sum_{i=1}^{p}b_{i}\right)\left(\prod_{i=1}^{p}b_{i}\right)}\right]+\sum_{i=1}^{p}\left(b_{i}-a_{i}\right)E\left(X_{i}\right)\\ \; & \;\;\;+E\left[\log\left\{1-\exp\left[-a_{p+1}\min\left(X_{1},\ldots ,X_{p}\right)\right]\right\}\right]\\ \; & \;\;\;-E\left[\log\left\{1-\exp\left[-b_{p+1}\min\left(X_{1},\ldots ,X_{p}\right)\right]\right\}\right]. \end{array}$$

Using the series expansion for log(1+z), we can express (16) as

$$\begin{array}{cc} \; & KLD=\log\left[\frac{b_{p+1}\left(\sum_{i=1}^{p}a_{i}\right)\left(\prod_{i=1}^{p}a_{i}\right)}{a_{p+1}\left(\sum_{i=1}^{p}b_{i}\right)\left(\prod_{i=1}^{p}b_{i}\right)}\right]+\sum_{i=1}^{p}\left(b_{i}-a_{i}\right)E\left(X_{i}\right)\\ \; & \;\;\;-\sum_{k=1}^{\infty }\frac{E\left\{\exp\left[-ka_{p+1}\min\left(X_{1},\ldots ,X_{p}\right)\right]\right\}}{k}\\ \; & \;\;\;+\sum_{k=1}^{\infty }\frac{E\left\{\exp\left[-kb_{p+1}\min\left(X_{1},\ldots ,X_{p}\right)\right]\right\}}{k}. \end{array}$$

Hence, the required.

### 4.13. Proof for Section 2.12

The corresponding KLD can be expressed as

$$\begin{array}{cc} KLD\; & =\log\left[\frac{\kappa_{1}^{\frac{p}{2}-1}}{\kappa_{2}^{\frac{p}{2}-1}}\frac{I_{\frac{p}{2}-1}\left(\kappa_{2}\right)}{I_{\frac{p}{2}-1}\left(\kappa_{1}\right)}\right]+\left(\kappa_{1}\mu_{1}^{T}-\kappa_{2}\mu_{2}^{T}\right)E\left(X\right)\\ \; & =\log\left[\frac{\kappa_{1}^{\frac{p}{2}-1}}{\kappa_{2}^{\frac{p}{2}-1}}\frac{I_{\frac{p}{2}-1}\left(\kappa_{2}\right)}{I_{\frac{p}{2}-1}\left(\kappa_{1}\right)}\right]+\left(\kappa_{1}\mu_{1}^{T}-\kappa_{2}\mu_{2}^{T}\right)\mu_{1}. \end{array}$$

Hence, the required.

### 4.14. Proof for Section 3.1

The corresponding KLD can be expressed as

(17) $$\begin{array}{c} KLD=\log\left[\frac{{\left|\Omega \right|}^{c+d-a-b}B_{p}\left(c,d\right)}{B_{p}\left(a,b\right)}\right]+\left(a-c\right)E\left[\log\left|\Omega -X\right|\right]+\left(b-d\right)E\left[\log\left|X\right|\right]. \end{array}$$

The expectations in (17) can be calculated as

$$\begin{array}{c} E\left[\log\left|\Omega -X\right|\right]={\left.\frac{\partial }{\partial \alpha }\left\{\int \frac{{\left|\Omega -x\right|}^{\alpha +a-\frac{p+1}{2}}{\left|x\right|}^{b-\frac{p+1}{2}}}{{\left|\Omega \right|}^{a+b}B_{p}\left(a,b\right)}dx\right\}\right|}_{\alpha =0}={\left.\frac{\partial }{\partial \alpha }\left\{\frac{{\left|\Omega \right|}^{\alpha }B_{p}\left(\alpha +a,b\right)}{B_{p}\left(a,b\right)}\right\}\right|}_{\alpha =0} \end{array}$$

and

$$\begin{array}{c} E\left[\log\left|X\right|\right]={\left.\frac{\partial }{\partial \alpha }\left\{\int \frac{{\left|\Omega -x\right|}^{a-\frac{p+1}{2}}{\left|x\right|}^{\alpha +b-\frac{p+1}{2}}}{{\left|\Omega \right|}^{a+b}B_{p}\left(a,b\right)}dx\right\}\right|}_{\alpha =0}={\left.\frac{\partial }{\partial \alpha }\left\{\frac{{\left|\Omega \right|}^{\alpha }B_{p}\left(a,\alpha +b\right)}{B_{p}\left(a,b\right)}\right\}\right|}_{\alpha =0}. \end{array}$$

Hence, the required.

### 4.15. Proof for Section 3.2

The corresponding KLD can be expressed as

(18) $$\begin{array}{cc} \; & KLD=\log\left[\frac{B_{p}\left(b_{1},\ldots ,b_{n};b_{n+1}\right)}{B_{p}\left(a_{1},\ldots ,a_{n};a_{n+1}\right)}\right]+\left(a_{n+1}-b_{n+1}\right)E\left[\log\left|I_{p}-\sum_{i=1}^{n}X_{i}\right|\right]\\ \; & \;\;\;+\sum_{i=1}^{n}\left(a_{i}-b_{i}\right)E\left[\log\left|X_{i}\right|\right]. \end{array}$$

It is easy to show that

$$\begin{array}{c} E\left[\log\left|X_{i}\right|\right]={\left.\frac{\partial }{\partial \alpha }\frac{B_{p}\left(a_{1},\ldots ,a_{i}+\alpha ,\ldots ,a_{n};a_{n+1}\right)}{B_{p}\left(a_{1},\ldots ,a_{i},\ldots ,a_{n};a_{n+1}\right)}\right|}_{\alpha =0} \end{array}$$

and

$$\begin{array}{c} E\left[\log\left|I_{p}-\sum_{i=1}^{n}X_{i}\right|\right]={\left.\frac{\partial }{\partial \alpha }\frac{B_{p}\left(a_{1},\ldots ,a_{n};a_{n+1}+\alpha \right)}{B_{p}\left(a_{1},\ldots ,a_{n};a_{n+1}\right)}\right|}_{\alpha =0}, \end{array}$$

so (18) reduces to the required.

### 4.16. Proof for Section 3.3

The corresponding KLD can be expressed as

(19) $$\begin{array}{cc} \; & KLD=\log\left[\frac{d^{pc}\Gamma_{p}\left(c\right)}{b^{pa}\Gamma_{p}\left(a\right)}\frac{{\left|\Sigma_{2}\right|}^{c}}{{\left|\Sigma_{1}\right|}^{a}}\right]+\left(a-c\right)E\left[\log\left|X\right|\right]+\mathrm{tr}\;\left[-\frac{1}{b}\Sigma_{1}^{-1}E\left(X\right)\right]\\ \; & \;\;\;-\mathrm{tr}\;\left[-\frac{1}{d}\Sigma_{2}^{-1}E\left(X\right)\right]. \end{array}$$

The first expectation in (19) can be calculated as

$$\begin{array}{cc} E\left[\log\left|X\right|\right] & =\frac{{\left|\Sigma_{1}\right|}^{-a}}{b^{ap}\Gamma_{p}\left(a\right)}{\left.\frac{\partial }{\partial \alpha }\left\{\int {\left|x\right|}^{\alpha +a-\frac{p+1}{2}}\exp\left[\mathrm{tr}\;\left(-\frac{1}{b}\Sigma_{1}^{-1}x\right)\right]\right\}\right|}_{\alpha =0}\\ \; & =\frac{1}{\Gamma_{p}\left(a\right)}{\left.\frac{\partial }{\partial \alpha }\left[{\left|\Sigma_{1}\right|}^{\alpha }b^{p\alpha }\Gamma_{p}\left(a+\alpha \right)\right]\right|}_{\alpha =0}. \end{array}$$

Since $E\left(X\right)=2a\Sigma_{1}$, the second and third terms in (19) are equal to

$$\begin{array}{c} \mathrm{tr}\;\left[-\frac{1}{b}\Sigma_{1}^{-1}E\left(X\right)\right]=\frac{2ap}{b} \end{array}$$

and

$$\begin{array}{c} \mathrm{tr}\;\left[-\frac{1}{d}\Sigma_{2}^{-1}E\left(X\right)\right]=\mathrm{tr}\;\left[-\frac{2a}{d}\Sigma_{2}^{-1}\Sigma_{1}\right], \end{array}$$

respectively. Hence, the required.

### 4.17. Proof for Section 3.4

The corresponding KLD can be expressed as

(20) $$\begin{array}{cc} \; & KLD=\log\left[\frac{B_{p}\left(d,e\right){\;}_{2}F_{1}\left(d,f;d+e;-B\right)}{B_{p}\left(a,b\right){\;}_{2}F_{1}\left(a,c;a+b;-B\right)}\right]+\left(a-d\right)E\left[\log\left|X\right|\right]+\left(b-e\right)E\left[\log\left|I_{p}-X\right|\right]\\ \; & \;\;\;+\left(f-c\right)E\left[\log\left|I_{p}+BX\right|\right]. \end{array}$$

The expectations in (20) can be easily calculated as

$$\begin{array}{c} E\left[\log\left|X\right|\right]={\left.\frac{\partial }{\partial \alpha }\frac{B_{p}\left(a+\alpha ,b\right){\;}_{2}F_{1}\left(a+\alpha ,c;a+b+\alpha ;-B\right)}{B_{p}\left(a,b\right){\;}_{2}F_{1}\left(a,c;a+b;-B\right)}\right|}_{\alpha =0}, \end{array}$$

$$\begin{array}{c} E\left[\log\left|I_{p}-X\right|\right]={\left.\frac{\partial }{\partial \alpha }\frac{B_{p}\left(a,b+\alpha \right){\;}_{2}F_{1}\left(a,c;a+b+\alpha ;-B\right)}{B_{p}\left(a,b\right){\;}_{2}F_{1}\left(a,c;a+b;-B\right)}\right|}_{\alpha =0} \end{array}$$

and

$$\begin{array}{c} E\left[\log\left|I_{p}+BX\right|\right]={\left.\frac{\partial }{\partial \alpha }\frac{{\;}_{2}F_{1}\left(a,c-\alpha ;a+b;-B\right)}{{\;}_{2}F_{1}\left(a,c;a+b;-B\right)}\right|}_{\alpha =0}. \end{array}$$

Hence, the required.

### 4.18. Proof for Section 3.5

The corresponding KLD can be expressed as

(21) $$\begin{array}{c} KLD=\log\left[\frac{{\left|\Omega \right|}^{a-c}B_{p}\left(c,d\right)}{B_{p}\left(a,b\right)}\right]+\left(c+d-a-b\right)E\left[\log\left|\Omega +X\right|\right]+\left(b-d\right)E\left[\log\left|X\right|\right]. \end{array}$$

The expectations in (21) can be calculated as

$$\begin{array}{c} E\left[\log\left|\Omega +X\right|\right]={\left.\frac{\partial }{\partial \alpha }\left\{\int \frac{{\left|\Omega +x\right|}^{\alpha -a-b}{\left|x\right|}^{b-\frac{p+1}{2}}}{{\left|\Omega \right|}^{-a}B_{p}\left(a,b\right)}dx\right\}\right|}_{\alpha =0}={\left.\frac{\partial }{\partial \alpha }\left\{\frac{{\left|\Omega \right|}^{\alpha }B_{p}\left(a-\alpha ,b\right)}{B_{p}\left(a,b\right)}\right\}\right|}_{\alpha =0} \end{array}$$

and

$$\begin{array}{c} E\left[\log\left|X\right|\right]={\left.\frac{\partial }{\partial \alpha }\left\{\int \frac{{\left|\Omega +x\right|}^{-a-b}{\left|x\right|}^{\alpha +b-\frac{p+1}{2}}}{{\left|\Omega \right|}^{-a}B_{p}\left(a,b\right)}dx\right\}\right|}_{\alpha =0}={\left.\frac{\partial }{\partial \alpha }\left\{\frac{{\left|\Omega \right|}^{\alpha }B_{p}\left(a-\alpha ,\alpha +b\right)}{B_{p}\left(a,b\right)}\right\}\right|}_{\alpha =0}. \end{array}$$

Hence, the required.

### 4.19. Proof for Section 3.6

The corresponding KLD can be expressed as

(22) $$\begin{array}{cc} \; & KLD=\log\left[\frac{d^{pc}\Gamma_{p}\left(c\right)}{b^{pa}\Gamma_{p}\left(a\right)}\frac{{\left|\Sigma_{1}\right|}^{a}}{{\left|\Sigma_{2}\right|}^{c}}\right]+\left(c-a\right)E\left[\log\left|X\right|\right]+\frac{1}{d}\mathrm{tr}\;\left[\Sigma_{2}E\left(X^{-1}\right)\right]\\ \; & \;\;\;-\frac{1}{b}\mathrm{tr}\;\left[\Sigma_{1}E\left(X^{-1}\right)\right]. \end{array}$$

The first expectation in (22) can be calculated as

$$\begin{array}{cc} E\left[\log\left|X\right|\right] & =\frac{{\left|\Sigma_{1}\right|}^{a}}{b^{ap}\Gamma_{p}\left(a\right)}{\left.\frac{\partial }{\partial \alpha }\left\{\int {\left|x\right|}^{\alpha -a-\frac{p+1}{2}}\exp\left[\mathrm{tr}\;\left(-\frac{1}{b}\Sigma_{1}x\right)\right]\right\}\right|}_{\alpha =0}\\ \; & =\frac{1}{\Gamma_{p}\left(a\right)}{\left.\frac{\partial }{\partial \alpha }\left[\frac{{\left|\Sigma_{1}\right|}^{\alpha }\Gamma_{p}\left(a-\alpha \right)}{b^{p\alpha }}\right]\right|}_{\alpha =0}. \end{array}$$

Since $E\left(X^{-1}\right)=2a\Sigma_{1}^{-1}$, the second and third terms in (22) are equal to

$$\begin{array}{c} \mathrm{tr}\;\left[\Sigma_{2}E\left(X^{-1}\right)\right]=2a\;\mathrm{tr}\;\left[\Sigma_{2}\Sigma_{1}^{-1}\right] \end{array}$$

and

$$\begin{array}{c} \mathrm{tr}\;\left[\Sigma_{1}E\left(X^{-1}\right)\right]=2ap, \end{array}$$

respectively. Hence, the required.

### 4.20. Proof for Section 3.7

The corresponding KLD can be expressed as

(23) $$\begin{array}{cc} \; & KLD=\log\left[\frac{B_{p}\left(c,d\right){\;}_{1}F_{1}\left(c;c+d;-B_{2}\right)}{B_{p}\left(a,b\right){\;}_{1}F_{1}\left(a,a+b;-B_{1}\right)}\right]+\left(a-c\right)E\left[\log\left|X\right|\right]+\left(b-d\right)E\left[\log\left|I_{p}-X\right|\right]\\ \; & \;\;\;+\mathrm{tr}\;\left[\left(B_{2}-B_{1}\right)E\left(X\right)\right]. \end{array}$$

The expectations in (23) can be easily calculated as

$$\begin{array}{c} E\left[\log\left|X\right|\right]={\left.\frac{\partial }{\partial \alpha }\frac{B_{p}\left(a+\alpha ,b\right){\;}_{1}F_{1}\left(a+\alpha ;a+b+\alpha ;-B_{1}\right)}{B_{p}\left(a,b\right){\;}_{1}F_{1}\left(a;a+b;-B_{1}\right)}\right|}_{\alpha =0}, \end{array}$$

$$\begin{array}{c} E\left[\log\left|I_{p}-X\right|\right]={\left.\frac{\partial }{\partial \alpha }\frac{B_{p}\left(a,b+\alpha \right){\;}_{1}F_{1}\left(a;a+b+\alpha ;-B_{1}\right)}{B_{p}\left(a,b\right){\;}_{1}F_{1}\left(a;a+b;-B_{1}\right)}\right|}_{\alpha =0} \end{array}$$

and

$$\begin{array}{c} E\left(X\right)={\left.\frac{\partial }{\partial z}\frac{{\;}_{1}F_{1}\left(a;a+b;z-B_{1}\right)}{{\;}_{1}F_{1}\left(a;a+b;-B_{1}\right)}\right|}_{z=0}. \end{array}$$

Hence, the required.

### 4.21. Proof for Section 3.8

The corresponding KLD can be expressed as

(24) $$\begin{array}{cc} \; & KLD=\log\left[\frac{\Gamma_{p}\left(c\right)\;\Psi_{1}\left(c;c-d+\frac{p+1}{2};B_{2}\right)}{\Gamma_{p}\left(a\right)\;\Psi_{1}\left(a;a-b+\frac{p+1}{2};B_{1}\right)}\right]+\left(a-c\right)E\left[\log\left|X\right|\right]+\left(d-b\right)E\left[\log\left|I_{p}+X\right|\right]\\ \; & \;\;\;+\mathrm{tr}\;\left[\left(B_{2}-B_{1}\right)E\left(X\right)\right]. \end{array}$$

The expectations in (24) can be easily calculated as

$$\begin{array}{c} E\left[\log\left|X\right|\right]={\left.\frac{\partial }{\partial \alpha }\frac{\Gamma_{p}\left(a+\alpha \right)\;\Psi_{1}\left(a+\alpha ;a-b+\alpha +\frac{p+1}{2};B_{1}\right)}{\Gamma_{p}\left(a\right)\;\Psi_{1}\left(a;a-b+\frac{p+1}{2};B_{1}\right)}\right|}_{\alpha =0}, \end{array}$$

$$\begin{array}{c} E\left[\log\left|I_{p}+X\right|\right]={\left.\frac{\partial }{\partial \alpha }\frac{\Psi_{1}\left(a;a-b+\alpha +\frac{p+1}{2};B_{1}\right)}{\Psi_{1}\left(a;a-b+\frac{p+1}{2};B_{1}\right)}\right|}_{\alpha =0} \end{array}$$

and

$$\begin{array}{c} E\left(X\right)={\left.\frac{\partial }{\partial z}\frac{\Psi_{1}\left(a;a-b+\frac{p+1}{2};B_{1}-z\right)}{\Psi_{1}\left(a;a-b+\frac{p+1}{2};B_{1}\right)}\right|}_{z=0}. \end{array}$$

Hence, the required.

### 4.22. Proof for Section 3.9

The corresponding KLD can be expressed as

(25) $$\begin{array}{cc} \; & KLD=\log\left[\frac{{\left|V_{2}\right|}^{\frac{n}{2}}{\left|U_{2}\right|}^{\frac{p}{2}}}{{\left|V_{1}\right|}^{\frac{n}{2}}{\left|U_{1}\right|}^{\frac{p}{2}}}\right]+\frac{1}{2}E\left\{\mathrm{tr}\;\left[V_{2}^{-1}{\left(X-M_{2}\right)}^{T}U_{2}^{-1}\left(X-M_{2}\right)\right]\right\}\\ \; & \;\;\;-\frac{1}{2}E\left\{\mathrm{tr}\;\left[V_{1}^{-1}{\left(X-M_{1}\right)}^{T}U_{1}^{-1}\left(X-M_{1}\right)\right]\right\}. \end{array}$$

The second expectation in (25) can be expressed as

$$\begin{array}{c} \mathrm{tr}\;\left\{V_{1}^{-1}E\left[{\left(X-M_{1}\right)}^{T}\left(X-M_{1}\right)\right]U_{1}^{-1}\right\}=\mathrm{tr}\;\left(U_{1}\right)\;\mathrm{tr}\;\left(U_{1}^{-1}\right). \end{array}$$

The first expectation in (25) can be expressed as

$$\begin{array}{cc} \; & \mathrm{tr}\;\left\{V_{2}^{-1}E\left[{\left(X-M_{2}\right)}^{T}\left(X-M_{2}\right)\right]U_{2}^{-1}\right\}\\ \; & =\mathrm{tr}\;\left[V_{2}^{-1}E\left(X^{T}X-X^{T}M_{2}-M_{2}^{T}X+M_{2}^{T}M_{2}\right)U_{2}^{-1}\right]\\ \; & =\mathrm{tr}\;\left\{V_{2}^{-1}\left[E\left(X^{T}X\right)-E\left(X^{T}M_{2}\right)-E\left(M_{2}^{T}X\right)+M_{2}^{T}M_{2}\right]U_{2}^{-1}\right\}\\ \; & =\mathrm{tr}\;\left(U_{1}\right)\;\mathrm{tr}\;\left(V_{2}^{-1}V_{1}U_{2}^{-1}\right)+\mathrm{tr}\;\left(V_{2}^{-1}M_{1}^{T}M_{1}U_{2}^{-1}\right)-\mathrm{tr}\;\left(V_{2}^{-1}M_{1}^{T}M_{2}U_{2}^{-1}\right)\\ \; & \;\;\;-\mathrm{tr}\;\left(V_{2}^{-1}M_{2}^{T}M_{1}U_{2}^{-1}\right)+\mathrm{tr}\;\left(V_{2}^{-1}M_{2}^{T}M_{2}U_{2}^{-1}\right). \end{array}$$

Hence, the required.

### 4.23. Proof for Section 3.10

The corresponding KLD can be expressed as

(26) $$\begin{array}{cc} \; & KLD=\log\left[\frac{C(a)}{C(b)}\right]+\left(a-b\right)E\left[\log\left|X\right|I\left\{0_{p}<X\leq B\right\}\right]\\ \; & \;\;\;+\left(a-b\right)E\left[\log\left|I_{p}-X\right|I\left\{B<X\leq I_{p}\right\}\right]\\ \; & \;\;\;+\left(b-a\right){\left|B\right|}^{\frac{p+1}{2}}\log\left|B\right|B_{p}\left(a,\frac{p+1}{2}\right)\\ \; & \;\;\;+\left(b-a\right){\left|I_{p}-B\right|}^{\frac{p+1}{2}}\log\left|I_{p}-B\right|B_{p}\left(\frac{p+1}{2},a\right). \end{array}$$

The expectations in (26) can be easily calculated as

$$\begin{array}{c} E\left[\log\left|X\right|I\left\{0_{p}<X\leq B\right\}\right]={\left.\frac{\partial }{\partial \alpha }\left\{{\left|B\right|}^{\alpha +\frac{p+1}{2}}B_{p}\left(a+\alpha ,\frac{p+1}{2}\right)\right\}\right|}_{\alpha =0} \end{array}$$

and

$$\begin{array}{c} E\left[\log\left|I_{p}-X\right|I\left\{B<X\leq I_{p}\right\}\right]={\left.\frac{\partial }{\partial \alpha }\left\{{\left|I_{p}-B\right|}^{\alpha +\frac{p+1}{2}}B_{p}\left(\frac{p+1}{2},a+\alpha \right)\right\}\right|}_{\alpha =0}. \end{array}$$

Hence, the required.

## Data Availability

No new data were created or analyzed in this study. Data sharing is not applicable to this article.

## Appendix A. Special Functions

The following special functions are used in the paper: the gamma function defined by

$$\begin{array}{c} \Gamma \left(a\right)=\int_{0}^{\infty }t^{a-1}\exp\left(-t\right)dt \end{array}$$

for a>0; the digamma function is defined by

$$\begin{array}{c} \psi \left(a\right)=\frac{d\log\Gamma (a)}{da} \end{array}$$

for a>0; the beta function is defined by

$$\begin{array}{c} B\left(a,b\right)=\int_{0}^{1}t^{a-1}{\left(1-t\right)}^{b-1}dt \end{array}$$

for a>0 and b>0; the type I Dirichlet density is defined by

$$\begin{array}{c} B\left(a_{1},\ldots ,a_{n};a_{n+1}\right)=\int_{0\leq t_{1}+\cdots +t_{n}\leq 1}t_{1}^{a_{1}-1}\cdots t_{n}^{a_{n}-1}{\left(1-\sum_{i=1}^{n}t_{i}\right)}^{a_{n+1}-1}dt_{1}\cdots dt_{n} \end{array}$$

for $a_{j}>0$, j=1,2,…,k+1; the modified Bessel function of the first kind of order ν defined by

$$\begin{array}{c} I_{\nu }\left(x\right)=\sum_{k=0}^{\infty }\frac{1}{\Gamma (k+\nu +1)k!}{\left(\frac{x}{2}\right)}^{2k+\nu } \end{array}$$

for k+ν+1≠0,−1,−2,…; the matrix-variate gamma function is defined by

$$\begin{array}{c} \Gamma_{p}\left(\alpha \right)=\int {\left|x\right|}^{\alpha -\frac{p+1}{2}}\exp\left[-\mathrm{tr}\;(x)\right]dx \end{array}$$

for x a p×p positive definite matrix and $a>\frac{p-1}{2}$; the matrix-variate beta function is defined by

$$\begin{array}{c} B_{p}\left(\alpha ,\beta \right)=\int {\left|I_{p}-x\right|}^{\alpha -\frac{p+1}{2}}{\left|x\right|}^{\beta -\frac{p+1}{2}}dx \end{array}$$

for x a p×p positive definite matrix, $I_{p}-x$ a p×p positive definite matrix, $\alpha >\frac{p-1}{2}$ and $\beta >\frac{p-1}{2}$; the matrix-variate type I Dirichlet density is defined by

$$\begin{array}{c} B_{p}\left(a_{1},\ldots ,a_{n};a_{n+1}\right)=\int {\left|x_{1}\right|}^{a_{1}-p}\cdots {\left|x_{n}\right|}^{a_{n}-p}{\left|I_{p}-\sum_{i=1}^{n}x_{i}\right|}^{a_{n+1}-p}dx_{1}\cdots dx_{n} \end{array}$$

for $x_{i}$, i=1,2,…,n and $I_{p}-\sum_{i=1}^{n}x_{i}$ being p×p positive definite matrices, and $a_{j}>\frac{p-1}{2}$, j=1,2,…,n+1; the matrix-variate confluent hypergeometric function is defined by

$$\begin{array}{c} {\;}_{1}F_{1}\left(a;b;X\right)=\sum_{k=0}^{\infty }\sum_{\kappa }\frac{{\left(a\right)}_{\kappa }}{{\left(b\right)}_{\kappa }}\frac{C_{\kappa }\left(X\right)}{k!} \end{array}$$

for X a p×p positive definite matrix and provided that $\Gamma_{p}\left(\alpha \right)$ and $\Gamma_{p}\left(\beta \right)$ exist; the matrix-variate Gauss hypergeometric function is defined by

$$\begin{array}{c} {\;}_{2}F_{1}\left(a,b;c;X\right)=\sum_{k=0}^{\infty }\sum_{\kappa }\frac{{\left(a\right)}_{\kappa }{\left(b\right)}_{\kappa }}{{\left(c\right)}_{\kappa }}\frac{C_{\kappa }\left(X\right)}{k!} \end{array}$$

for X a p×p positive definite matrix and provided that $\Gamma_{p}\left(a\right)$, $\Gamma_{p}\left(b\right)$ and $\Gamma_{p}\left(c\right)$ exist, where $C_{\kappa }\left(X\right)$ denotes the zonal polynomial of the p×p symmetric matrix X corresponding to the ordered partition $\kappa =\left(k_{1},\ldots ,k_{p}\right)$ with $k_{1}\geq \cdots \geq k_{p}\geq 0$ and $k_{1}+\cdots +k_{p}=k$, $\sum_{\kappa }$ denotes summation over all such partitions κ, and

$$\begin{array}{c} {\left(a\right)}_{\kappa }=\prod_{i=1}^{p}{\left(a-\frac{i-1}{2}\right)}_{k_{i}}, \end{array}$$

where ${\left(a\right)}_{0}=1$ and ${\left(a\right)}_{k}=a\left(a+1\right)\cdot \left(a+k-1\right)$.
